# Supplementary material for: Social distancing is a social dilemma game played by every individual against his/her population
Source: PLoS One. 2021 Aug 2;16(8):e0255543. doi: 10.1371/journal.pone.0255543 (PMC8328347; doi:10.1371/journal.pone.0255543)
Supplement: S1 File — Simulation for a small single population with 10 social activities to form a Petersen’s diagram. (PDF) [file pone.0255543.s001.pdf]

**S1 File. Simulation Results 1:** Simulation on a small single population, with the activities connected as a Petersen's graph

Population size: 100
Activities: {1,2,3,4,5,6,7,8,9,10}
Contact values: {4,4,4,4,4,1,1,1,1,1}

Connectivity:

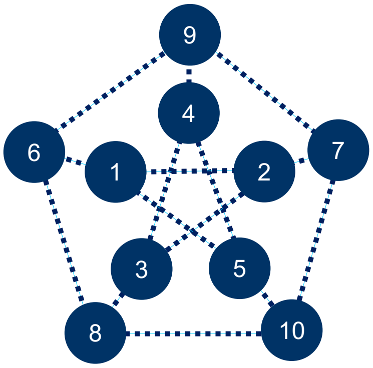


S1 Fig 1. Connections among activities.

Number of test runs: 10 with random initial strategies for all the individuals.
Number of generations: 100

S1 Table 1: Distancing Strategies at Equilibrium in 10 Test Runs

| Act | Test 1 | Test 2 | Test 3 | Test 4 | Test 5 | Test 6 | Test 7 | Test 8 | Test 9 | Test 10 |
|---|---|---|---|---|---|---|---|---|---|---|
| 1 | 0.111111 | 0.040366 | 0.100000 | 0.100000 | 0.111111 | 0.000000 | 0.000000 | 0.000000 | 0.000000 | 0.000000 |
| 2 | 0.000000 | 0.070745 | 0.000000 | 0.000000 | 0.000000 | 0.100000 | 0.000000 | 0.000000 | 0.000000 | 0.000000 |
| 3 | 0.000000 | 0.000000 | 0.100000 | 0.100000 | 0.000000 | 0.000000 | 0.100000 | 0.000000 | 0.000000 | 0.000000 |
| 4 | 0.000000 | 0.000000 | 0.000000 | 0.000000 | 0.000000 | 0.100000 | 0.000000 | 0.000002 | 0.000004 | 0.000000 |
| 5 | 0.000000 | 0.000000 | 0.000000 | 0.000000 | 0.000000 | 0.000000 | 0.100000 | 0.111109 | 0.111106 | 0.111111 |
| 6 | 0.000000 | 0.000000 | 0.000000 | 0.000000 | 0.000000 | 0.400000 | 0.400000 | 0.039938 | 0.011825 | 0.000179 |
| 7 | 0.000041 | 0.000000 | 0.000000 | 0.000000 | 0.000000 | 0.000000 | 0.399999 | 0.444444 | 0.444446 | 0.444445 |
| 8 | 0.444444 | 0.211448 | 0.000000 | 0.000000 | 0.443385 | 0.000000 | 0.000000 | 0.404506 | 0.432619 | 0.444266 |
| 9 | 0.444403 | 0.444443 | 0.400000 | 0.400000 | 0.444444 | 0.000000 | 0.000000 | 0.000000 | 0.000000 | 0.000000 |
| 10 | 0.000000 | 0.232999 | 0.400000 | 0.400000 | 0.001060 | 0.400000 | 0.000000 | 0.000000 | 0.000000 | 0.000000 |

Legends: Act – Activities; Rows – Frequencies for each activity; Columns – Frequencies for all activities.
