# Supplementary material for: Social distancing is a social dilemma game played by every individual against his/her population
Source: PLoS One. 2021 Aug 2;16(8):e0255543. doi: 10.1371/journal.pone.0255543 (PMC8328347; doi:10.1371/journal.pone.0255543)
Supplement: S2 File — Simulation for a small multi-population with 10 social activities to form a Petersen’s diagram. (PDF) [file pone.0255543.s002.pdf]

S2 File. Simulation Results 2: Simulation on small multi-populations, with the activities connected as a Petersen's graph

Population size: 100; Subpopulation 1: 50; Subpopulation 2: 50
Activities: {1,2,3,4,5,6,7,8,9,10}; Subpopulation 1: {1,2,3,4,5,6,7,9}; Subpopulation 2: {1,2,3,4,5,8,10}
Contact values: {4,4,4,4,4,1,1,1,1,1}

Connectivity:

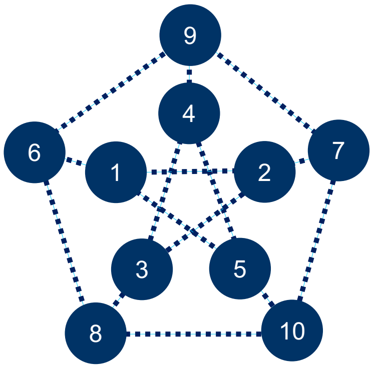

S2 Fig 1. Connections among activities.

Number of test runs: 10 with random initial strategies for all the individuals.
Number of generations: 100

S2 Table 1: Distancing Strategies at Equilibrium for Subpopulation 1 in 10 Test Runs

Act	Test 1	Test 2	Test 3	Test 4	Test 5	Test 6	Test 7	Test 8	Test 9	Test 10
1	0.111111	0.000000	0.000000	0.111279	0.111114	0.000000	0.000000	0.164139	0.000173	0.000000
2	0.000000	0.111111	0.111112	0.000000	0.000000	0.028763	0.109533	0.000000	0.000000	0.111016
3	0.000000	0.000000	0.000000	0.000000	0.000000	0.000000	0.000000	0.000000	0.000000	0.000092
4	0.000000	0.000000	0.000000	0.000000	0.000000	0.171237	0.090466	0.035850	0.000000	0.000000
5	0.000000	0.000000	0.000000	0.000000	0.000000	0.000000	0.000000	0.000000	0.110938	0.000000
6	0.000000	0.000614	0.000006	0.000002	0.000000	0.799999	0.800000	0.000011	0.000000	0.014209
7	0.000000	0.000000	0.000002	0.000000	0.000000	0.000000	0.000000	0.800000	0.888821	0.000000
8	0.000000	0.000000	0.000000	0.000000	0.000000	0.000000	0.000000	0.000000	0.000000	0.000000
9	0.888889	0.888275	0.888880	0.888718	0.888886	0.000001	0.000000	0.000000	0.000068	0.874683
10	0.000000	0.000000	0.000000	0.000000	0.000000	0.000000	0.000000	0.000000	0.000000	0.000000

Legend: Act – Activities; Rows – Frequencies for each activity; Columns – Frequencies for all activities.

S2 Table 2: Distancing Strategies at Equilibrium for Subpopulation 2 in 10 Test Runs

Act	Test 1	Test 2	Test 3	Test 4	Test 5	Test 6	Test 7	Test 8	Test 9	Test 10
1	0.111111	0.000000	0.000000	0.110946	0.111109	0.000000	0.000000	0.035855	0.000000	0.000000
2	0.000000	0.111112	0.111110	0.000000	0.000000	0.171237	0.090467	0.000000	0.000000	0.111114
3	0.000000	0.000000	0.000000	0.000000	0.000000	0.000000	0.000000	0.000000	0.000000	0.000000
4	0.000000	0.000000	0.000000	0.000000	0.000000	0.028762	0.109534	0.164152	0.000000	0.000000
5	0.000000	0.000000	0.000000	0.000000	0.000000	0.000000	0.000000	0.000000	0.111111	0.000000
6	0.000000	0.000000	0.000000	0.000000	0.000000	0.000000	0.000000	0.000000	0.000000	0.000000
7	0.000000	0.000000	0.000000	0.000000	0.000000	0.000000	0.000000	0.000000	0.000000	0.000000
8	0.888889	0.000006	0.000093	0.450744	0.762841	0.000000	0.000000	0.799993	0.888889	0.000000
9	0.000000	0.000000	0.000000	0.000000	0.000000	0.000000	0.000000	0.000000	0.000000	0.000000
10	0.000000	0.888883	0.888797	0.438310	0.126049	0.800001	0.800000	0.000000	0.000000	0.888886

Legend: Act – Activities; Rows – Frequencies for each activity; Columns – Frequencies for all activities.
