# Supplementary material for: Social distancing is a social dilemma game played by every individual against his/her population
Source: PLoS One. 2021 Aug 2;16(8):e0255543. doi: 10.1371/journal.pone.0255543 (PMC8328347; doi:10.1371/journal.pone.0255543)
Supplement: S3 File — Simulation for a single large population in the small university town. (PDF) [file pone.0255543.s003.pdf]

S3 File. Simulation Results 3: Simulation on a large single population resembling a university town

Population size: 850
Activities: {1, …, 85}

Contact values:

S3 Table 1: Contact Values for All the activity sites

Act	Con	Act	Con	Act	Con	Act	Con	Act	Con	Act	Con	Act	Con	Act	Con	Act	Con	Act	Con
1	6	10	6	19	2	28	4	37	1	46	4	55	2	64	4	73	4	82	4
2	6	11	6	20	2	29	1	38	1	47	4	56	2	65	4	74	4	83	4
3	6	12	6	21	2	30	1	39	1	48	4	57	2	66	4	75	4	84	4
4	6	13	6	22	2	31	1	40	1	49	2	58	2	67	4	76	2	85	4
5	6	14	2	23	2	32	1	41	1	50	2	59	2	68	4	77	2		
6	6	15	2	24	2	33	1	42	1	51	4	60	2	69	4	78	2		
7	6	16	2	25	4	34	1	43	1	52	4	61	2	70	4	79	2		
8	6	17	2	26	4	35	1	44	1	53	4	62	2	71	4	80	4		
9	6	18	2	27	4	36	1	45	4	54	4	63	2	72	4	81	4		

Legends: Act – Activities; Con – Contact values.

Connectivity: See S3 Fig 1.

Number of test runs: 10 with random initial strategies for all the individuals.
Number of generations: 400

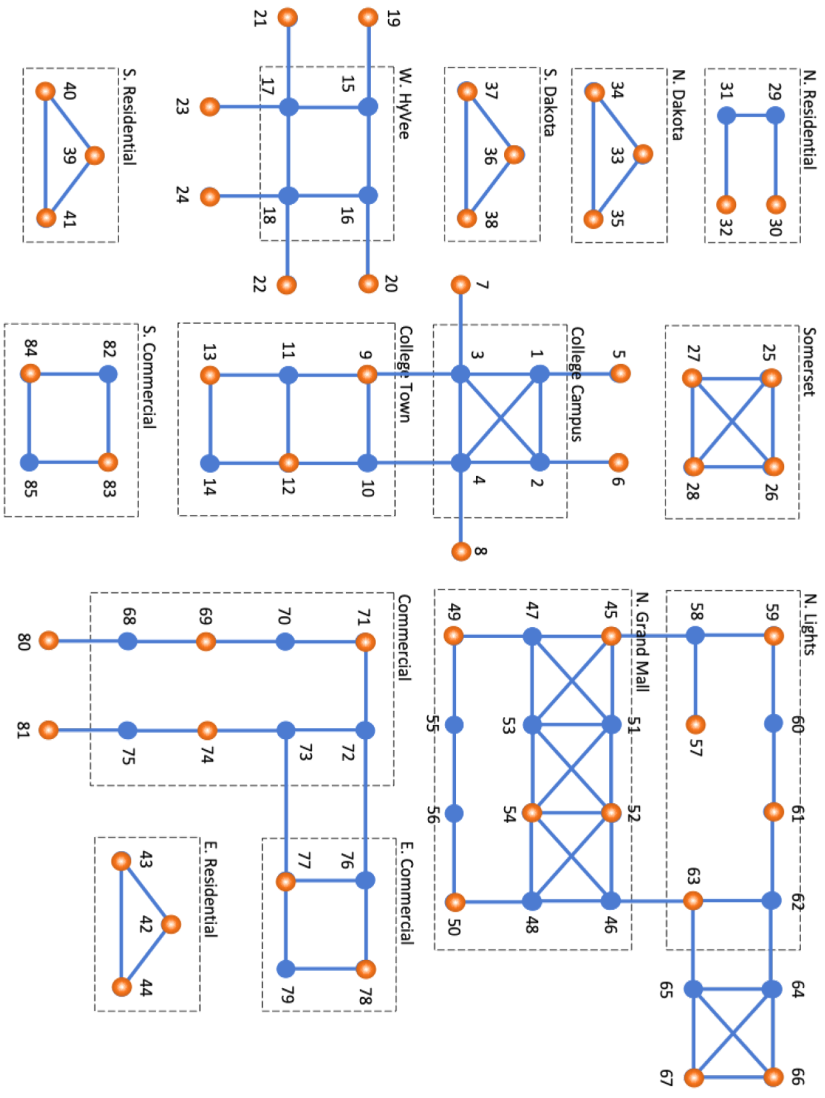

S3 Fig 1. Connections among activity sites. Activities in orange are those selected by a distancing strategy at equilibrium.

S3 Table 2: Distancing Strategies at Equilibrium in 10 Test Runs

Act	Test 1	Test 2	Test 3	Test 4	Test 5	Test 6	Test 7	Test 8	Test 9	Test 10
1	0.000000	0.000000	0.000007	0.000000	0.000000	0.000001	0.000000	0.000012	0.000002	0.000000
2	0.000000	0.000000	0.000000	0.000000	0.000000	0.000000	0.000000	0.000000	0.000000	0.000001
3	0.000000	0.000000	0.000000	0.000000	0.000000	0.000000	0.000000	0.000000	0.000000	0.000000
4	0.000000	0.000000	0.000000	0.000000	0.000000	0.000000	0.000000	0.000000	0.000000	0.000000
5	0.009852	0.009852	0.009846	0.009852	0.009852	0.009852	0.009852	0.009840	0.009851	0.009852
6	0.009852	0.009852	0.009852	0.009852	0.009852	0.009852	0.009852	0.009852	0.009852	0.009851
7	0.009852	0.009852	0.009852	0.009852	0.009852	0.009852	0.009852	0.009852	0.009852	0.009852
8	0.009852	0.009852	0.009852	0.009852	0.009852	0.009852	0.009852	0.009852	0.009852	0.009852
9	0.009852	0.009852	0.000000	0.000000	0.009852	0.009852	0.009852	0.000000	0.009852	0.000000
10	0.000000	0.000000	0.009852	0.009852	0.000000	0.000000	0.000000	0.009852	0.000000	0.009852
11	0.000000	0.000000	0.009852	0.009852	0.000000	0.000000	0.000000	0.009852	0.000000	0.009852
12	0.009852	0.009852	0.000000	0.000000	0.009852	0.009852	0.009852	0.000000	0.009852	0.000000
13	0.009852	0.009852	0.000000	0.000000	0.009852	0.009852	0.009852	0.000000	0.009852	0.000000
14	0.000000	0.000000	0.009852	0.009852	0.000000	0.000000	0.000000	0.009852	0.000000	0.009851
15	0.000118	0.000000	0.000000	0.000000	0.000000	0.000000	0.000000	0.000001	0.000000	0.000009
16	0.000000	0.000018	0.000018	0.000001	0.000013	0.000000	0.000008	0.000000	0.000000	0.000000
17	0.000000	0.000000	0.000000	0.000000	0.000000	0.000000	0.000000	0.000000	0.000000	0.000000
18	0.000000	0.000000	0.000000	0.000000	0.000000	0.000000	0.000000	0.000000	0.000000	0.000000
19	0.029439	0.029557	0.029557	0.029557	0.029557	0.029557	0.029557	0.029556	0.029557	0.029547
20	0.029557	0.029538	0.029539	0.029556	0.029544	0.029557	0.029549	0.029560	0.029556	0.029557
21	0.029557	0.029557	0.029557	0.029557	0.029557	0.029557	0.029557	0.029557	0.029557	0.029557
22	0.029557	0.029557	0.029557	0.029557	0.029557	0.029557	0.029557	0.029556	0.029557	0.029557
23	0.029557	0.029557	0.029557	0.029557	0.029557	0.029557	0.029557	0.029556	0.029557	0.029556
24	0.029557	0.029557	0.029557	0.029557	0.029557	0.029557	0.029557	0.029557	0.029557	0.029556
25	0.003801	0.003511	0.003859	0.003746	0.003826	0.003816	0.003515	0.003492	0.003624	0.003450
26	0.003476	0.003682	0.003706	0.003845	0.003486	0.003667	0.003648	0.003767	0.003683	0.003856
27	0.003642	0.003688	0.003565	0.003874	0.003624	0.003982	0.003902	0.003495	0.003772	0.003826
28	0.003859	0.003898	0.003648	0.003313	0.003842	0.003313	0.003714	0.004024	0.003699	0.003646
29	0.000021	0.000000	0.000000	0.000000	0.000000	0.000019	0.000000	0.000014	0.000000	0.000005
30	0.059092	0.059113	0.059113	0.059113	0.059113	0.059094	0.059113	0.059098	0.059113	0.059109
31	0.000000	0.000011	0.000000	0.000010	0.000000	0.000000	0.000007	0.000000	0.000013	0.000000
32	0.059113	0.059103	0.059113	0.059104	0.059113	0.059113	0.059106	0.059113	0.059100	0.059113
33	0.019707	0.019956	0.020047	0.019307	0.019050	0.019875	0.019418	0.019590	0.020081	0.019373
34	0.019925	0.019616	0.019452	0.020108	0.020184	0.019562	0.019629	0.020371	0.019437	0.020188
35	0.019481	0.019542	0.019615	0.019698	0.019879	0.019676	0.020066	0.019152	0.019595	0.019552
36	0.019544	0.019935	0.019679	0.019635	0.019586	0.019572	0.019400	0.019733	0.020244	0.019416
37	0.020088	0.019766	0.019816	0.019755	0.019585	0.019756	0.019633	0.019352	0.019735	0.019688
38	0.019481	0.019412	0.019617	0.019723	0.019942	0.019785	0.020080	0.020029	0.019134	0.020009
39	0.019293	0.019627	0.019844	0.020103	0.019639	0.019567	0.019461	0.020211	0.019923	0.019656
40	0.019626	0.020059	0.020009	0.019715	0.019344	0.019729	0.019997	0.019141	0.019490	0.019793
41	0.020195	0.019428	0.019260	0.019296	0.020130	0.019817	0.019655	0.019761	0.019701	0.019664
42	0.019149	0.019363	0.019473	0.020309	0.019663	0.020153	0.019423	0.019263	0.019586	0.019546
43	0.019873	0.019969	0.019883	0.019397	0.019345	0.019593	0.019889	0.020350	0.019662	0.019900
44	0.020092	0.019781	0.019758	0.019407	0.020106	0.019367	0.019802	0.019500	0.019865	0.019668
45	0.014778	0.014778	0.014778	0.014778	0.014778	0.014778	0.014778	0.014779	0.014778	0.014778

Legends: Act – Activities; Rows – Frequencies for each activity; Columns – Frequencies for all activities.

S3 Table 2: (Continue) Distancing Strategies at Equilibrium in 10 Test Runs

Act	Test 1	Test 2	Test 3	Test 4	Test 5	Test 6	Test 7	Test 8	Test 9	Test 10
46	0.000000	0.000000	0.000000	0.000000	0.000000	0.000000	0.000000	0.000000	0.000000	0.000000
47	0.000000	0.000000	0.000000	0.000000	0.000000	0.000000	0.000000	0.000000	0.000000	0.000000
48	0.000000	0.000000	0.000000	0.000000	0.000000	0.000000	0.000000	0.000000	0.000000	0.000000
49	0.029547	0.029549	0.029557	0.029557	0.029557	0.029556	0.029549	0.029557	0.029557	0.029556
50	0.029557	0.029557	0.029543	0.029534	0.029555	0.029557	0.029557	0.029554	0.029542	0.029560
51	0.000000	0.000000	0.000000	0.000000	0.000000	0.000000	0.000000	0.000000	0.000000	0.000000
52	0.007311	0.007840	0.007270	0.007004	0.007337	0.006891	0.007157	0.007676	0.006871	0.007733
53	0.000000	0.000000	0.000000	0.000000	0.000000	0.000000	0.000000	0.000000	0.000000	0.000000
54	0.007467	0.006939	0.007509	0.007774	0.007441	0.007888	0.007621	0.007102	0.007908	0.007045
55	0.000010	0.000007	0.000000	0.000000	0.000000	0.000000	0.000007	0.000000	0.000000	0.000000
56	0.000000	0.000000	0.000014	0.000023	0.000001	0.000000	0.000000	0.000002	0.000015	0.000000
57	0.029557	0.029557	0.029557	0.029557	0.029557	0.029557	0.029557	0.029557	0.029557	0.029557
58	0.000000	0.000000	0.000000	0.000000	0.000000	0.000000	0.000000	0.000000	0.000000	0.000000
59	0.029557	0.029557	0.029557	0.029557	0.029557	0.029557	0.029557	0.029557	0.029557	0.029557
60	0.000000	0.000000	0.000000	0.000000	0.000000	0.000000	0.000000	0.000000	0.000000	0.000000
61	0.029557	0.029557	0.029557	0.029557	0.029557	0.029557	0.029557	0.029556	0.029557	0.029557
62	0.000000	0.000000	0.000000	0.000000	0.000000	0.000000	0.000000	0.000000	0.000000	0.000000
63	0.029557	0.029557	0.029557	0.029557	0.029557	0.029557	0.029557	0.029557	0.029557	0.029557
64	0.000000	0.000002	0.000001	0.000002	0.000003	0.000002	0.000002	0.000001	0.000000	0.000006
65	0.000000	0.000000	0.000000	0.000000	0.000000	0.000000	0.000000	0.000000	0.000000	0.000000
66	0.006515	0.007517	0.007840	0.007389	0.007185	0.007447	0.007418	0.007563	0.006972	0.007187
67	0.008263	0.007259	0.006937	0.007388	0.007590	0.007330	0.007358	0.007214	0.007806	0.007586
68	0.000000	0.000000	0.000000	0.000000	0.000000	0.000000	0.000000	0.000000	0.000000	0.000000
69	0.014778	0.014778	0.014778	0.014778	0.004680	0.014778	0.014778	0.014778	0.014778	0.014778
70	0.000000	0.000000	0.000000	0.000000	0.010099	0.000000	0.000000	0.000000	0.000000	0.000000
71	0.014770	0.014778	0.014764	0.014778	0.000000	0.014778	0.014769	0.014778	0.014778	0.014778
72	0.000009	0.000000	0.000014	0.000000	0.014778	0.000000	0.000010	0.000000	0.000000	0.000000
73	0.000000	0.000026	0.000000	0.000013	0.000000	0.000005	0.000000	0.000004	0.000013	0.000002
74	0.014778	0.014752	0.014778	0.014765	0.014778	0.014773	0.014778	0.014775	0.014765	0.014776
75	0.000000	0.000000	0.000000	0.000000	0.000000	0.000000	0.000000	0.000000	0.000000	0.000000
76	0.000000	0.029557	0.000000	0.029557	0.000000	0.029557	0.000000	0.029556	0.029557	0.029557
77	0.029557	0.000000	0.029557	0.000000	0.029557	0.000000	0.029557	0.000000	0.000000	0.000000
78	0.029557	0.000000	0.029557	0.000000	0.029557	0.000000	0.029557	0.000000	0.000000	0.000000
79	0.000000	0.029557	0.000000	0.029557	0.000000	0.029557	0.000000	0.029556	0.029557	0.029557
80	0.014778	0.014778	0.014778	0.014778	0.014778	0.014778	0.014778	0.014778	0.014778	0.014778
81	0.014778	0.014778	0.014778	0.014778	0.014778	0.014778	0.014778	0.014778	0.014778	0.014778
82	0.000000	0.000000	0.014778	0.000000	0.014778	0.000000	0.014778	0.014778	0.014778	0.000000
83	0.014778	0.014778	0.000000	0.014778	0.000000	0.014778	0.000000	0.000000	0.000000	0.014778
84	0.014778	0.014778	0.000000	0.014778	0.000000	0.014778	0.000000	0.000000	0.000000	0.014778
85	0.000000	0.000000	0.014778	0.000000	0.014778	0.000000	0.014778	0.014778	0.014778	0.000000
86										
87										
88										
89										
90										

Legends: Act – Activities; Rows – Frequencies for each activity; Columns – Frequencies for all activities.
