# Supplementary material for: Social distancing is a social dilemma game played by every individual against his/her population
Source: PLoS One. 2021 Aug 2;16(8):e0255543. doi: 10.1371/journal.pone.0255543 (PMC8328347; doi:10.1371/journal.pone.0255543)
Supplement: S4 File — Simulation for a large multi-population in the small university town. (PDF) [file pone.0255543.s004.pdf]

S4 File. Simulation Results 4: Simulation on large multi-populations resembling a university town

Population size: 850
Subpopulation 1: 250; Subpopulation 2: 120; Subpopulation 3: 120; Subpopulation 4: 120; Subpopulation 5: 120; Subpopulation: 120

Activities: {1, …, 85}
Subpopulation 1: {1-24,45-56,}; Subpopulation 2: {1-8,25-32,45-85};
Subpopulation 3: {1-8,15-28,33-35,45-56,68-85}; Subpopulation 4: {1-8,15-28,36-38,45-56,68,85}
Subpopulation 5: {1-8,15-28,39-41,45-56,68-85}; Subpopulation 6: {1-8,25-28,42-85}

Contact values:

S4 Table 1: Contact Values for All the activity Sites

Act	Con	Act	Con	Act	Con	Act	Con	Act	Con	Act	Con	Act	Con	Act	Con	Act	Con	Act	Con
1	6	10	6	19	2	28	4	37	1	46	4	55	2	64	4	73	4	82	4
2	6	11	6	20	2	29	1	38	1	47	4	56	2	65	4	74	4	83	4
3	6	12	6	21	2	30	1	39	1	48	4	57	2	66	4	75	4	84	4
4	6	13	6	22	2	31	1	40	1	49	2	58	2	67	4	76	2	85	4
5	6	14	2	23	2	32	1	41	1	50	2	59	2	68	4	77	2		
6	6	15	2	24	2	33	1	42	1	51	4	60	2	69	4	78	2		
7	6	16	2	25	4	34	1	43	1	52	4	61	2	70	4	79	2		
8	6	17	2	26	4	35	1	44	1	53	4	62	2	71	4	80	4		
9	6	18	2	27	4	36	1	45	4	54	4	63	2	72	4	81	4		

Legends: Act – Activities; Con – Contact values.

Connectivity: See S4 Fig 1.

Number of test runs: 10 with random initial strategies for all the individuals.
Number of generations: 400

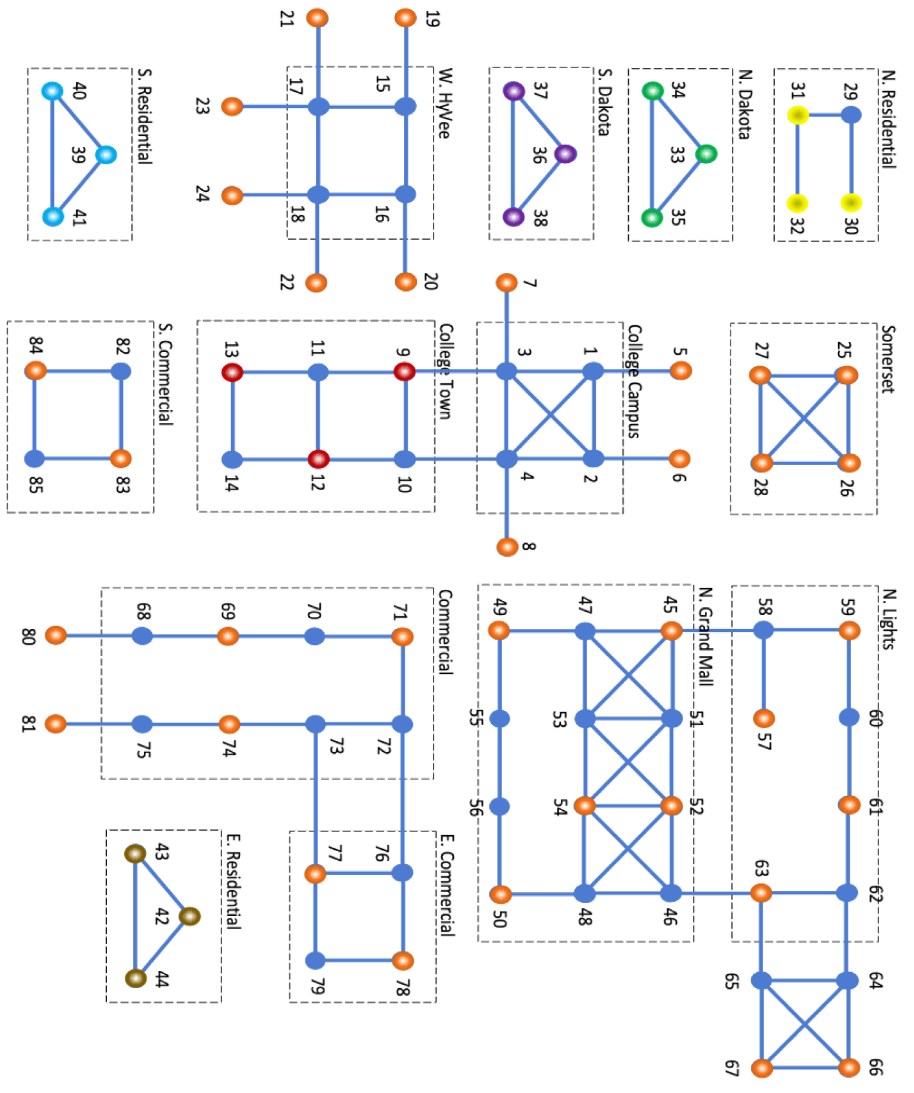

S4 Fig 1. Connections among activity sites. Activities in blue are those not selected by a distancing strategy at equilibrium. Other color code: red for Subpop 1; yellow for Subpop 2; green for Subpop 3; purple for Subpop 4; light blue for Subpop 5; brown for Subpop 6; orange for Mixed populations.

S4 Table 2: Distancing Strategies at Equilibrium from Test 1

Act	Subpop 1	Subpop 2	Subpop 3	Subpop 4	Subpop 5	Subpop 6
1	0.000000	0.000000	0.000000	0.000000	0.000000	0.000000
2	0.000000	0.000000	0.000000	0.000000	0.000000	0.000000
3	0.000000	0.000000	0.000000	0.000000	0.000000	0.000000
4	0.000000	0.000000	0.000000	0.000000	0.000000	0.000000
5	0.059100	0.000000	0.000001	0.000000	0.000002	0.000010
6	0.059028	0.000000	0.000003	0.000000	0.000000	0.000083
7	0.058773	0.000000	0.000050	0.000067	0.000223	0.000000
8	0.057771	0.000000	0.000018	0.000000	0.000028	0.001296
9	0.059113	0.000000	0.000000	0.000000	0.000000	0.000000
10	0.000000	0.000000	0.000000	0.000000	0.000000	0.000000
11	0.000000	0.000000	0.000000	0.000000	0.000000	0.000000
12	0.059113	0.000000	0.000000	0.000000	0.000000	0.000000
13	0.059113	0.000000	0.000000	0.000000	0.000000	0.000000
14	0.000000	0.000000	0.000000	0.000000	0.000000	0.000000
15	0.000004	0.000000	0.000005	0.000002	0.000011	0.000000
16	0.000000	0.000000	0.000000	0.000000	0.000000	0.000000
17	0.000000	0.000000	0.000000	0.000000	0.000000	0.000000
18	0.000000	0.000000	0.000000	0.000000	0.000000	0.000000
19	0.065714	0.000000	0.037796	0.036920	0.036886	0.000000
20	0.073708	0.000000	0.035533	0.035272	0.032827	0.000000
21	0.045614	0.000000	0.043437	0.036431	0.051858	0.000000
22	0.046421	0.000000	0.045359	0.032974	0.052586	0.000000
23	0.042893	0.000000	0.043121	0.035923	0.055403	0.000000
24	0.045392	0.000000	0.047954	0.034741	0.049252	0.000000
25	0.000000	0.000001	0.008149	0.002985	0.008078	0.003138
26	0.000000	0.000000	0.008013	0.001993	0.009881	0.001336
27	0.000000	0.000001	0.007220	0.003220	0.008690	0.002501
28	0.000000	0.000003	0.006878	0.003492	0.009914	0.003177
29	0.000000	0.000000	0.000000	0.000000	0.000000	0.000000
30	0.000000	0.354680	0.000000	0.000000	0.000000	0.000000
31	0.000000	0.000173	0.000000	0.000000	0.000000	0.000000
32	0.000000	0.354507	0.000000	0.000000	0.000000	0.000000
33	0.000000	0.000000	0.113215	0.000000	0.000000	0.000000
34	0.000000	0.000000	0.118777	0.000000	0.000000	0.000000
35	0.000000	0.000000	0.122688	0.000000	0.000000	0.000000
36	0.000000	0.000000	0.000000	0.121979	0.000000	0.000000
37	0.000000	0.000000	0.000000	0.121056	0.000000	0.000000
38	0.000000	0.000000	0.000000	0.111644	0.000000	0.000000
39	0.000000	0.000000	0.000000	0.000000	0.108951	0.000000
40	0.000000	0.000000	0.000000	0.000000	0.121131	0.000000
41	0.000000	0.000000	0.000000	0.000000	0.124598	0.000000
42	0.000000	0.000000	0.000000	0.000000	0.000000	0.107635
43	0.000000	0.000000	0.000000	0.000000	0.000000	0.115647
44	0.000000	0.000000	0.000000	0.000000	0.000000	0.131397
45	0.040829	0.001316	0.014658	0.003171	0.022279	0.006418

Legends: Act – Activities; Subpop – Subpopulation;
Columns – Frequencies on all activities for each subpopulation.

S4 Table 2 (Continue): Distancing Strategies at Equilibrium from Test 1

Act	Subpop 1	Subpop 2	Subpop 3	Subpop 4	Subpop 5	Subpop 6
46	0.000000	0.000000	0.000000	0.000000	0.000000	0.000000
47	0.000000	0.000000	0.000000	0.000000	0.000000	0.000000
48	0.000000	0.000000	0.000000	0.000000	0.000000	0.000000
49	0.100460	0.000002	0.011766	0.033909	0.021227	0.009972
50	0.096996	0.000005	0.010818	0.031799	0.026976	0.010746
51	0.000000	0.000000	0.000000	0.000000	0.000000	0.000000
52	0.019234	0.000000	0.001311	0.017415	0.006453	0.009477
53	0.000000	0.000000	0.000000	0.000000	0.000000	0.000000
54	0.010721	0.000000	0.001455	0.012143	0.004708	0.005752
55	0.000002	0.000000	0.000001	0.000000	0.000002	0.000000
56	0.000000	0.000000	0.000000	0.000000	0.000000	0.000000
57	0.000000	0.043988	0.000000	0.000000	0.000000	0.133352
58	0.000000	0.000000	0.000000	0.000000	0.000000	0.000000
59	0.000000	0.097246	0.000000	0.000000	0.000000	0.080094
60	0.000000	0.000000	0.000000	0.000000	0.000000	0.000000
61	0.000000	0.070764	0.000000	0.000000	0.000000	0.106576
62	0.000000	0.000000	0.000000	0.000000	0.000000	0.000000
63	0.000000	0.059247	0.000000	0.000000	0.000000	0.118093
64	0.000000	0.000000	0.000000	0.000000	0.000000	0.000005
65	0.000000	0.000000	0.000000	0.000000	0.000000	0.000000
66	0.000000	0.002527	0.000000	0.000000	0.000000	0.041239
67	0.000000	0.002146	0.000000	0.000000	0.000000	0.042752
68	0.000000	0.000000	0.000000	0.000000	0.000000	0.000000
69	0.000000	0.000001	0.033888	0.036071	0.012778	0.005932
70	0.000000	0.000000	0.000000	0.000000	0.000000	0.000000
71	0.000000	0.000003	0.030707	0.038521	0.012134	0.007304
72	0.000000	0.000000	0.000000	0.000000	0.000000	0.000000
73	0.000000	0.000000	0.000000	0.000000	0.000000	0.000000
74	0.000000	0.000000	0.028322	0.036024	0.011088	0.013235
75	0.000000	0.000000	0.000000	0.000000	0.000000	0.000000
76	0.000000	0.000000	0.000000	0.000000	0.000000	0.000000
77	0.000000	0.007765	0.054375	0.019362	0.089995	0.005843
78	0.000000	0.005527	0.056114	0.056306	0.052029	0.007364
79	0.000000	0.000000	0.000000	0.000000	0.000000	0.000000
80	0.000000	0.000027	0.023341	0.039636	0.017551	0.008114
81	0.000000	0.000071	0.026847	0.039384	0.014244	0.008124
82	0.000000	0.000000	0.000000	0.000000	0.000000	0.000000
83	0.000000	0.000000	0.032133	0.030142	0.018670	0.007725
84	0.000000	0.000000	0.036050	0.027415	0.019543	0.005662
85	0.000000	0.000000	-0.000000	-0.000000	0.000000	0.000000
86						
87						
88						
89						
90						

Legends: Act – Activities; Subpop – Subpopulation;
Columns – Frequencies on all activities for each subpopulation.

S4 Table 3: Distancing Strategies at Equilibrium from Test 2

Act	Subpop 1	Subpop 2	Subpop 3	Subpop 4	Subpop 5	Subpop 6
1	0.000000	0.000000	0.000000	0.000000	0.000000	0.000000
2	0.000000	0.000000	0.000000	0.000000	0.000000	0.000000
3	-0.000000	0.000000	0.000000	0.000000	0.000000	0.000000
4	0.000000	0.000000	0.000000	0.000000	0.000000	0.000000
5	0.059055	0.000001	0.000026	0.000004	0.000027	0.000001
6	0.058356	0.000000	0.000418	0.000003	0.000023	0.000313
7	0.058758	0.000000	0.000059	0.000003	0.000029	0.000264
8	0.059112	0.000000	0.000001	0.000000	0.000000	0.000000
9	0.059113	0.000000	0.000000	0.000000	0.000000	0.000000
10	0.000000	0.000000	0.000000	0.000000	0.000000	0.000000
11	0.000000	0.000000	0.000000	0.000000	0.000000	0.000000
12	0.059113	0.000000	0.000000	0.000000	0.000000	0.000000
13	0.059113	0.000000	0.000000	0.000000	0.000000	0.000000
14	0.000000	0.000000	0.000000	0.000000	0.000000	0.000000
15	0.000000	0.000000	0.000001	0.000001	0.000008	0.000000
16	0.000000	0.000000	0.000000	0.000000	0.000000	0.000000
17	0.000000	0.000000	0.000000	0.000000	0.000000	0.000000
18	0.000000	0.000000	0.000000	0.000000	0.000000	0.000000
19	0.083152	0.000000	0.019095	0.039913	0.035170	0.000000
20	0.082539	0.000000	0.018750	0.046663	0.029388	0.000000
21	0.028452	0.000000	0.050201	0.038870	0.059816	0.000000
22	0.025967	0.000000	0.047608	0.040636	0.063128	0.000000
23	0.035397	0.000000	0.050841	0.034117	0.056985	0.000000
24	0.023794	0.000000	0.051569	0.036919	0.065057	0.000000
25	0.000000	0.000002	0.007492	0.003406	0.003940	0.004003
26	0.000000	0.000016	0.008285	0.003264	0.005447	0.004880
27	0.000000	0.000007	0.007451	0.004323	0.005582	0.006219
28	0.000000	0.000019	0.008662	0.004330	0.007160	0.004181
29	0.000000	0.000189	0.000000	0.000000	0.000000	0.000000
30	0.000000	0.354491	0.000000	0.000000	0.000000	0.000000
31	0.000000	0.000000	0.000000	0.000000	0.000000	0.000000
32	0.000000	0.354680	0.000000	0.000000	0.000000	0.000000
33	0.000000	0.000000	0.128933	0.000000	0.000000	0.000000
34	0.000000	0.000000	0.119766	0.000000	0.000000	0.000000
35	0.000000	0.000000	0.105981	0.000000	0.000000	0.000000
36	0.000000	0.000000	0.000000	0.110604	0.000000	0.000000
37	0.000000	0.000000	0.000000	0.113190	0.000000	0.000000
38	0.000000	0.000000	0.000000	0.130886	0.000000	0.000000
39	0.000000	0.000000	0.000000	0.000000	0.109031	0.000000
40	0.000000	0.000000	0.000000	0.000000	0.128358	0.000000
41	0.000000	0.000000	0.000000	0.000000	0.117291	0.000000
42	0.000000	0.000000	0.000000	0.000000	0.000000	0.115138
43	0.000000	0.000000	0.000000	0.000000	0.000000	0.114097
44	0.000000	0.000000	0.000000	0.000000	0.000000	0.125445
45	0.033992	0.003008	0.008561	0.007767	0.016753	0.018589

Legends: Act – Activities; Subpop – Subpopulation;
Columns – Frequencies on all activities for each subpopulation.

S4 Table 3 (Continue): Distancing Strategies at Equilibrium from Test 2

Act	Subpop 1	Subpop 2	Subpop 3	Subpop 4	Subpop 5	Subpop 6
46	0.000000	0.000000	0.000000	0.000000	0.000000	0.000000
47	0.000000	0.000000	0.000000	0.000000	0.000000	0.000000
48	0.000000	0.000000	0.000000	0.000000	0.000000	0.000000
49	0.122640	0.000010	0.005122	0.017218	0.028872	0.003478
50	0.117451	0.000003	0.012277	0.013563	0.029448	0.004505
51	0.000000	0.000000	0.000000	0.000000	0.000000	0.000000
52	0.016857	0.000000	0.003262	0.011405	0.010936	0.003012
53	0.000000	0.000000	0.000000	0.000000	0.000000	0.000000
54	0.017124	0.000000	0.001988	0.014555	0.007444	0.002088
55	0.000000	0.000000	0.000000	0.000000	0.000000	0.000000
56	0.000014	0.000003	0.000002	0.000004	0.000068	0.000002
57	0.000000	0.056994	0.000000	0.000000	0.000000	0.120346
58	0.000000	0.000000	0.000000	0.000000	0.000000	0.000000
59	0.000000	0.082011	0.000000	0.000000	0.000000	0.095329
60	0.000000	0.000000	0.000000	0.000000	0.000000	0.000000
61	0.000000	0.084310	0.000000	0.000000	0.000000	0.093030
62	0.000000	0.000000	0.000000	0.000000	0.000000	0.000000
63	0.000000	0.043570	0.000000	0.000000	0.000000	0.133770
64	0.000000	0.000001	0.000000	0.000000	0.000000	0.000005
65	0.000000	0.000000	0.000000	0.000000	0.000000	0.000000
66	0.000000	0.000381	0.000000	0.000000	0.000000	0.041798
67	0.000000	0.000462	0.000000	0.000000	0.000000	0.046024
68	0.000000	0.000000	0.000000	0.000000	0.000000	0.000000
69	0.000000	0.000000	0.036117	0.038241	0.010731	0.003581
70	0.000000	0.000000	0.000000	0.000000	0.000000	0.000000
71	0.000000	0.000010	0.032156	0.037085	0.014010	0.005409
72	0.000000	0.000000	0.000000	0.000000	0.000000	0.000000
73	0.000000	0.000000	0.000001	0.000001	0.000000	0.000000
74	0.000000	0.000000	0.034022	0.041809	0.009709	0.003128
75	0.000000	0.000000	0.000000	0.000000	0.000000	0.000000
76	0.000000	0.011632	0.052479	0.019829	0.063665	0.029735
77	0.000000	0.000000	0.000000	0.000000	0.000000	0.000000
78	0.000000	0.000000	0.000000	0.000000	0.000000	0.000000
79	0.000000	0.007878	0.056460	0.048324	0.056632	0.008046
80	0.000000	0.000131	0.032179	0.035682	0.015428	0.005250
81	0.000000	0.000190	0.033401	0.033047	0.018637	0.003395
82	0.000000	0.000000	0.000000	0.000000	0.000000	0.000000
83	0.000000	0.000001	0.036050	0.034710	0.015315	0.002594
84	0.000000	0.000001	0.030787	0.039625	0.015911	0.002345
85	0.000000	0.000000	0.000000	0.000000	-0.000000	0.000000
86						
87						
88						
89						
90						

Legends: Act – Activities; Subpop – Subpopulation;
Columns – Frequencies on all activities for each subpopulation.

S4 Table 4: Distancing Strategies at Equilibrium from Test 3

Act	Subpop 1	Subpop 2	Subpop 3	Subpop 4	Subpop 5	Subpop 6
1	0.000000	0.000000	0.000001	0.000001	0.000000	0.000000
2	0.000000	0.000000	0.000000	0.000000	0.000000	0.000000
3	0.000000	0.000000	0.000000	0.000000	0.000000	0.000000
4	0.000000	0.000000	0.000000	0.000000	0.000000	0.000000
5	0.059000	0.000000	0.000026	0.000021	0.000063	0.000000
6	0.056833	0.000000	0.000533	0.000012	0.000502	0.001232
7	0.059111	0.000000	0.000000	0.000000	0.000002	0.000000
8	0.054397	0.000000	0.000381	0.000038	0.002202	0.002096
9	0.059113	0.000000	0.000000	0.000000	0.000000	0.000000
10	0.000000	0.000000	0.000000	0.000000	0.000000	0.000000
11	0.000000	0.000000	0.000000	0.000000	0.000000	0.000000
12	0.059113	0.000000	0.000000	0.000000	0.000000	0.000000
13	0.059113	0.000000	0.000000	0.000000	0.000000	0.000000
14	0.000000	0.000000	0.000000	0.000000	0.000000	0.000000
15	0.000013	0.000000	0.000003	0.000038	0.000036	0.000000
16	0.000000	0.000000	0.000000	0.000000	0.000000	0.000000
17	0.000000	0.000000	0.000000	0.000000	0.000000	0.000000
18	0.000000	0.000000	0.000000	0.000000	0.000000	0.000000
19	0.068759	0.000000	0.029013	0.043618	0.035860	0.000000
20	0.072099	0.000000	0.030157	0.045126	0.029958	0.000000
21	0.048530	0.000000	0.039144	0.040498	0.049167	0.000000
22	0.037104	0.000000	0.050706	0.039867	0.049662	0.000000
23	0.049524	0.000000	0.039255	0.037831	0.050729	0.000000
24	0.044789	0.000000	0.048498	0.034345	0.049707	0.000000
25	0.000000	0.000000	0.011314	0.004397	0.004865	0.003934
26	0.000000	0.000000	0.009570	0.004252	0.003889	0.003267
27	0.000000	0.000000	0.009527	0.003572	0.003189	0.004859
28	0.000000	0.000000	0.009484	0.005427	0.003236	0.003887
29	0.000000	0.000000	0.000000	0.000000	0.000000	0.000000
30	0.000000	0.354680	0.000000	0.000000	0.000000	0.000000
31	0.000000	0.000008	0.000000	0.000000	0.000000	0.000000
32	0.000000	0.354672	0.000000	0.000000	0.000000	0.000000
33	0.000000	0.000000	0.121268	0.000000	0.000000	0.000000
34	0.000000	0.000000	0.114031	0.000000	0.000000	0.000000
35	0.000000	0.000000	0.119380	0.000000	0.000000	0.000000
36	0.000000	0.000000	0.000000	0.128991	0.000000	0.000000
37	0.000000	0.000000	0.000000	0.112316	0.000000	0.000000
38	0.000000	0.000000	0.000000	0.113372	0.000000	0.000000
39	0.000000	0.000000	0.000000	0.000000	0.117202	0.000000
40	0.000000	0.000000	0.000000	0.000000	0.119648	0.000000
41	0.000000	0.000000	0.000000	0.000000	0.117830	0.000000
42	0.000000	0.000000	0.000000	0.000000	0.000000	0.131018
43	0.000000	0.000000	0.000000	0.000000	0.000000	0.112639
44	0.000000	0.000000	0.000000	0.000000	0.000000	0.111023
45	0.039618	0.000974	0.011037	0.020961	0.007432	0.008649

Legends: Act – Activities; Subpop – Subpopulation;
Columns – Frequencies on all activities for each subpopulation.

S4 Table 4 (Continue): Distancing Strategies at Equilibrium from Test 3

Act	Subpop 1	Subpop 2	Subpop 3	Subpop 4	Subpop 5	Subpop 6
46	0.000000	0.000000	0.000000	0.000000	0.000000	0.000000
47	0.000000	0.000000	0.000000	0.000000	0.000000	0.000000
48	0.000000	0.000000	0.000000	0.000000	0.000000	0.000000
49	0.097602	0.000001	0.013012	0.022365	0.036556	0.007582
50	0.110896	0.000003	0.011527	0.013514	0.033115	0.008285
51	0.000000	0.000000	0.000000	0.000000	0.000000	0.000000
52	0.014048	0.000000	0.006906	0.011052	0.009817	0.005650
53	0.000000	0.000000	0.000000	0.000000	0.000000	0.000000
54	0.010184	0.000000	0.005174	0.013922	0.007408	0.004510
55	0.000153	0.000002	0.000007	0.000042	0.000018	0.000000
56	0.000000	0.000000	0.000000	0.000000	0.000000	0.000000
57	0.000000	0.050637	0.000000	0.000000	0.000000	0.126702
58	0.000000	0.000000	0.000000	0.000000	0.000000	0.000000
59	0.000000	0.080997	0.000000	0.000000	0.000000	0.096343
60	0.000000	0.000000	0.000000	0.000000	0.000000	0.000000
61	0.000000	0.081173	0.000000	0.000000	0.000000	0.096167
62	0.000000	0.000000	0.000000	0.000000	0.000000	0.000000
63	0.000000	0.058413	0.000000	0.000000	0.000000	0.118927
64	0.000000	0.000000	0.000000	0.000000	0.000000	0.000005
65	0.000000	0.000000	0.000000	0.000000	0.000000	0.000000
66	0.000000	0.000003	0.000000	0.000000	0.000000	0.046763
67	0.000000	0.000001	0.000000	0.000000	0.000000	0.041899
68	0.000000	0.000000	0.000000	0.000000	0.000000	0.000000
69	0.000000	0.000000	0.030872	0.033114	0.018991	0.005694
70	0.000000	0.000000	0.000000	0.000000	0.000000	0.000000
71	0.000000	0.000000	0.031550	0.030510	0.018126	0.008484
72	0.000000	0.000000	0.000000	0.000000	0.000000	0.000000
73	0.000000	0.000000	0.000000	0.000000	0.000000	0.000000
74	0.000000	0.000000	0.027951	0.028912	0.025331	0.006475
75	0.000000	0.000000	0.000000	0.000000	0.000000	0.000000
76	0.000000	0.007174	0.052164	0.045990	0.060432	0.011580
77	0.000000	0.000000	0.000000	0.000000	0.000000	0.000000
78	0.000000	0.000000	0.000000	0.000000	0.000000	0.000000
79	0.000000	0.011142	0.068955	0.042862	0.039200	0.015181
80	0.000000	0.000034	0.031355	0.024698	0.022170	0.010412
81	0.000000	0.000086	0.034055	0.021641	0.026869	0.006019
82	0.000000	0.000000	0.000000	0.000000	0.000000	0.000000
83	0.000000	0.000000	0.024071	0.036202	0.027987	0.000410
84	0.000000	0.000000	0.019072	0.040489	0.028802	0.000307
85	0.000000	0.000000	0.000000	0.000000	0.000000	0.000000
86						
87						
88						
89						
90						

Legends: Act – Activities; Subpop – Subpopulation;
Columns – Frequencies on all activities for each subpopulation.

S4 Table 5: Distancing Strategies at Equilibrium from Test 4

Act	Subpop 1	Subpop 2	Subpop 3	Subpop 4	Subpop 5	Subpop 6
1	0.000002	0.000000	0.000000	0.000000	0.000000	0.000000
2	0.000000	0.000000	0.000000	0.000000	0.000000	0.000000
3	0.000000	0.000000	0.000000	0.000000	0.000000	0.000000
4	0.000000	0.000000	0.000000	0.000000	0.000000	0.000000
5	0.059099	0.000000	0.000000	0.000000	0.000001	0.000012
6	0.059065	0.000000	0.000000	0.000000	0.000000	0.000048
7	0.059055	0.000000	0.000000	0.000000	0.000000	0.000058
8	0.058794	0.000000	0.000034	0.000001	0.000266	0.000019
9	0.059113	0.000000	0.000000	0.000000	0.000000	0.000000
10	0.000000	0.000000	0.000000	0.000000	0.000000	0.000000
11	0.000000	0.000000	0.000000	0.000000	0.000000	0.000000
12	0.059113	0.000000	0.000000	0.000000	0.000000	0.000000
13	0.059113	0.000000	0.000000	0.000000	0.000000	0.000000
14	0.000000	0.000000	0.000000	0.000000	0.000000	0.000000
15	0.000000	0.000000	0.000000	0.000000	0.000000	0.000000
16	0.000003	0.000000	0.000002	0.000009	0.000014	0.000000
17	0.000000	0.000000	0.000000	0.000000	0.000000	0.000000
18	0.000000	0.000000	0.000000	0.000000	0.000000	0.000000
19	0.064898	0.000000	0.035566	0.044045	0.032831	0.000000
20	0.064100	0.000000	0.040837	0.032903	0.039472	0.000000
21	0.037253	0.000000	0.048334	0.034122	0.057631	0.000000
22	0.039119	0.000000	0.053484	0.028639	0.056098	0.000000
23	0.042847	0.000000	0.048101	0.027825	0.058567	0.000000
24	0.044166	0.000000	0.048629	0.033203	0.051342	0.000000
25	0.000000	0.000000	0.005364	0.004274	0.008037	0.004640
26	0.000000	0.000000	0.006282	0.005446	0.008428	0.003390
27	0.000000	0.000000	0.005726	0.004885	0.007541	0.003637
28	0.000000	0.000000	0.006610	0.005353	0.006011	0.003044
29	0.000000	0.000762	0.000000	0.000000	0.000000	0.000000
30	0.000000	0.353918	0.000000	0.000000	0.000000	0.000000
31	0.000000	0.000000	0.000000	0.000000	0.000000	0.000000
32	0.000000	0.354680	0.000000	0.000000	0.000000	0.000000
33	0.000000	0.000000	0.113872	0.000000	0.000000	0.000000
34	0.000000	0.000000	0.113140	0.000000	0.000000	0.000000
35	0.000000	0.000000	0.127668	0.000000	0.000000	0.000000
36	0.000000	0.000000	0.000000	0.121118	0.000000	0.000000
37	0.000000	0.000000	0.000000	0.119710	0.000000	0.000000
38	0.000000	0.000000	0.000000	0.113852	0.000000	0.000000
39	0.000000	0.000000	0.000000	0.000000	0.118787	0.000000
40	0.000000	0.000000	0.000000	0.000000	0.116694	0.000000
41	0.000000	0.000000	0.000000	0.000000	0.119199	0.000000
42	0.000000	0.000000	0.000000	0.000000	0.000000	0.106619
43	0.000000	0.000000	0.000000	0.000000	0.000000	0.126005
44	0.000000	0.000000	0.000000	0.000000	0.000000	0.122056
45	0.036512	0.007482	0.006410	0.007672	0.023918	0.006676

Legends: Act – Activities; Subpop – Subpopulation;
Columns – Frequencies on all activities for each subpopulation.

S4 Table 5 (Continue): Distancing Strategies at Equilibrium from Test 4

Act	Subpop 1	Subpop 2	Subpop 3	Subpop 4	Subpop 5	Subpop 6
46	0.000000	0.000000	0.000000	0.000000	0.000000	0.000000
47	0.000000	0.000000	0.000000	0.000000	0.000000	0.000000
48	0.000000	0.000000	0.000000	0.000000	0.000000	0.000000
49	0.110563	0.000001	0.004909	0.013514	0.039044	0.009310
50	0.109666	0.000009	0.004300	0.023692	0.035444	0.004191
51	0.000000	0.000000	0.000000	0.000000	0.000000	0.000000
52	0.020784	0.000000	0.002428	0.014211	0.003167	0.005170
53	0.000000	0.000000	0.000000	0.000000	0.000000	0.000000
54	0.016729	0.000000	0.002707	0.013931	0.004328	0.005215
55	0.000000	0.000000	0.000000	0.000000	0.000000	0.000000
56	0.000006	0.000002	0.000000	0.000009	0.000021	0.000000
57	0.000000	0.059074	0.000000	0.000000	0.000000	0.118266
58	0.000000	0.000000	0.000000	0.000000	0.000000	0.000000
59	0.000000	0.063724	0.000000	0.000000	0.000000	0.113616
60	0.000000	0.000000	0.000000	0.000000	0.000000	0.000000
61	0.000000	0.089085	0.000000	0.000000	0.000000	0.088255
62	0.000000	0.000000	0.000000	0.000000	0.000000	0.000000
63	0.000000	0.050745	0.000000	0.000000	0.000000	0.126595
64	0.000000	0.000001	0.000000	0.000000	0.000000	0.000004
65	0.000000	0.000000	0.000000	0.000000	0.000000	0.000000
66	0.000000	0.001150	0.000000	0.000000	0.000000	0.044458
67	0.000000	0.001024	0.000000	0.000000	0.000000	0.042033
68	0.000000	0.000000	0.000000	0.000000	0.000000	0.000000
69	0.000000	0.000003	0.029831	0.035238	0.015739	0.007859
70	0.000000	0.000000	0.000000	0.000000	0.000000	0.000000
71	0.000000	0.000001	0.029145	0.036397	0.019674	0.003453
72	0.000000	0.000000	0.000001	0.000000	0.000000	0.000000
73	0.000000	0.000000	0.000000	0.000000	0.000000	0.000000
74	0.000000	0.000006	0.024354	0.031231	0.024397	0.008683
75	0.000000	0.000000	0.000000	0.000000	0.000000	0.000000
76	0.000000	0.000000	0.000000	0.000000	0.000000	0.000000
77	0.000000	0.008901	0.055324	0.049614	0.052993	0.010506
78	0.000000	0.008915	0.062433	0.054870	0.034711	0.016412
79	0.000000	0.000000	0.000000	0.000000	0.000000	0.000000
80	0.000000	0.000370	0.031289	0.031803	0.018657	0.006552
81	0.000000	0.000103	0.027887	0.036095	0.018186	0.006399
82	0.000000	0.000000	0.000000	0.000000	0.000000	0.000000
83	0.000000	0.000000	0.032188	0.038375	0.013816	0.004291
84	0.000000	0.000044	0.033145	0.037966	0.014985	0.002530
85	0.000000	0.000000	0.000000	0.000000	0.000000	0.000000
86						
87						
88						
89						
90						

Legends: Act – Activities; Subpop – Subpopulation;
Columns – Frequencies on all activities for each subpopulation.

S4 Table 6: Distancing Strategies at Equilibrium from Test 5

Act	Subpop 1	Subpop 2	Subpop 3	Subpop 4	Subpop 5	Subpop 6
1	0.000000	0.000000	0.000000	0.000000	0.000000	0.000000
2	0.000000	0.000000	0.000000	0.000000	0.000000	0.000000
3	0.000000	0.000000	0.000000	0.000000	0.000000	0.000000
4	0.000000	0.000000	0.000000	0.000000	0.000000	0.000000
5	0.059058	0.000000	0.000000	0.000000	0.000006	0.000049
6	0.059092	0.000000	0.000008	0.000000	0.000002	0.000011
7	0.059087	0.000000	0.000024	0.000000	0.000001	0.000000
8	0.058295	0.000000	0.000001	0.000000	0.000061	0.000756
9	0.059113	0.000000	0.000000	0.000000	0.000000	0.000000
10	0.000000	0.000000	0.000000	0.000000	0.000000	0.000000
11	0.000000	0.000000	0.000000	0.000000	0.000000	0.000000
12	0.059113	0.000000	0.000000	0.000000	0.000000	0.000000
13	0.059113	0.000000	0.000000	0.000000	0.000000	0.000000
14	0.000000	0.000000	0.000000	0.000000	0.000000	0.000000
15	0.000000	0.000000	0.000000	0.000000	0.000000	0.000000
16	0.000018	0.000000	0.000001	0.000059	0.000051	0.000000
17	0.000000	0.000000	0.000000	0.000000	0.000000	0.000000
18	0.000000	0.000000	0.000000	0.000000	0.000000	0.000000
19	0.059254	0.000000	0.026749	0.054357	0.036980	0.000000
20	0.068470	0.000000	0.034840	0.043676	0.030225	0.000000
21	0.041307	0.000000	0.041449	0.047579	0.047005	0.000000
22	0.045861	0.000000	0.041308	0.041205	0.048967	0.000000
23	0.036207	0.000000	0.043482	0.045270	0.052381	0.000000
24	0.051528	0.000000	0.038301	0.044148	0.043363	0.000000
25	0.000000	0.000000	0.009728	0.004118	0.009800	0.003029
26	0.000000	0.000000	0.007481	0.002870	0.007384	0.001969
27	0.000000	0.000000	0.008237	0.003667	0.006281	0.003196
28	0.000000	0.000000	0.007046	0.003253	0.006865	0.003746
29	0.000000	0.000000	0.000000	0.000000	0.000000	0.000000
30	0.000000	0.354680	0.000000	0.000000	0.000000	0.000000
31	0.000000	0.000516	0.000000	0.000000	0.000000	0.000000
32	0.000000	0.354164	0.000000	0.000000	0.000000	0.000000
33	0.000000	0.000000	0.122667	0.000000	0.000000	0.000000
34	0.000000	0.000000	0.120560	0.000000	0.000000	0.000000
35	0.000000	0.000000	0.111453	0.000000	0.000000	0.000000
36	0.000000	0.000000	0.000000	0.128833	0.000000	0.000000
37	0.000000	0.000000	0.000000	0.108580	0.000000	0.000000
38	0.000000	0.000000	0.000000	0.117266	0.000000	0.000000
39	0.000000	0.000000	0.000000	0.000000	0.112396	0.000000
40	0.000000	0.000000	0.000000	0.000000	0.117907	0.000000
41	0.000000	0.000000	0.000000	0.000000	0.124377	0.000000
42	0.000000	0.000000	0.000000	0.000000	0.000000	0.109513
43	0.000000	0.000000	0.000000	0.000000	0.000000	0.124195
44	0.000000	0.000000	0.000000	0.000000	0.000000	0.120972
45	0.028857	0.000920	0.009583	0.004116	0.028586	0.016608

Legends: Act – Activities; Subpop – Subpopulation;
Columns – Frequencies on all activities for each subpopulation.

S4 Table 6 (Continue): Distancing Strategies at Equilibrium from Test 5

Act	Subpop 1	Subpop 2	Subpop 3	Subpop 4	Subpop 5	Subpop 6
46	0.000000	0.000000	0.000000	0.000000	0.000000	0.000000
47	0.000000	0.000000	0.000000	0.000000	0.000000	0.000000
48	0.000000	0.000000	0.000000	0.000000	0.000000	0.000000
49	0.110836	0.000002	0.003891	0.033179	0.026516	0.002889
50	0.113671	0.000000	0.001130	0.025701	0.033898	0.002939
51	0.000000	0.000000	0.000000	0.000000	0.000000	0.000000
52	0.013019	0.000000	0.002714	0.009888	0.007158	0.010208
53	0.000000	0.000000	0.000000	0.000000	0.000000	0.000000
54	0.018097	0.000000	0.002773	0.007960	0.007942	0.008911
55	0.000001	0.000001	0.000000	0.000003	0.000022	0.000000
56	0.000000	0.000000	0.000000	0.000000	0.000000	0.000000
57	0.000000	0.036069	0.000000	0.000000	0.000000	0.141271
58	0.000000	0.000000	0.000000	0.000000	0.000000	0.000000
59	0.000000	0.077066	0.000000	0.000000	0.000000	0.100274
60	0.000000	0.000000	0.000000	0.000000	0.000000	0.000000
61	0.000000	0.089340	0.000000	0.000000	0.000000	0.087999
62	0.000000	0.000000	0.000000	0.000000	0.000000	0.000000
63	0.000000	0.065053	0.000000	0.000000	0.000000	0.112287
64	0.000000	0.000000	0.000000	0.000000	0.000000	0.000000
65	0.000000	0.000000	0.000000	0.000000	0.000000	0.000000
66	0.000000	0.003706	0.000000	0.000000	0.000000	0.041338
67	0.000000	0.003836	0.000000	0.000000	0.000000	0.039789
68	0.000000	0.000000	0.000000	0.000000	0.000000	0.000000
69	0.000000	0.000000	0.044344	0.024009	0.017087	0.003230
70	0.000000	0.000000	0.000000	0.000000	0.000000	0.000000
71	0.000000	0.000000	0.031207	0.034403	0.016236	0.006816
72	0.000000	0.000000	0.000002	0.000005	0.000001	0.000000
73	0.000000	0.000000	0.000000	0.000000	0.000000	0.000000
74	0.000000	0.000001	0.032170	0.031036	0.016035	0.009428
75	0.000000	0.000000	0.000000	0.000000	0.000000	0.000000
76	0.000000	0.000000	0.000000	0.000000	0.000000	0.000000
77	0.000000	0.008161	0.042384	0.030316	0.082966	0.013512
78	0.000000	0.006341	0.060338	0.049672	0.052051	0.008938
79	0.000000	0.000000	0.000000	0.000000	0.000000	0.000000
80	0.000000	0.000104	0.041821	0.021551	0.017893	0.007301
81	0.000000	0.000036	0.041346	0.020980	0.017608	0.008700
82	0.000000	0.000001	0.042909	0.025542	0.014765	0.005453
83	0.000000	0.000000	0.000000	0.000000	0.000000	0.000000
84	0.000000	0.000000	0.000000	0.000000	0.000000	0.000000
85	0.000000	0.000001	0.030054	0.036757	0.017185	0.004673
86						
87						
88						
89						
90						

Legends: Act – Activities; Subpop – Subpopulation;
Columns – Frequencies on all activities for each subpopulation.

S4 Table 7: Distancing Strategies at Equilibrium from Test 6

Act	Subpop 1	Subpop 2	Subpop 3	Subpop 4	Subpop 5	Subpop 6
1	0.000000	0.000000	0.000000	0.000000	0.000000	0.000000
2	0.000000	0.000000	0.000000	0.000000	0.000000	0.000000
3	0.000000	0.000000	0.000000	0.000000	0.000000	0.000000
4	0.000000	0.000000	0.000000	0.000000	0.000000	0.000000
5	0.059057	0.000000	0.000009	0.000000	0.000047	0.000000
6	0.059111	0.000000	0.000002	0.000000	0.000000	0.000000
7	0.059081	0.000000	0.000016	0.000002	0.000014	0.000000
8	0.058875	0.000000	0.000152	0.000002	0.000066	0.000018
9	0.059113	0.000000	0.000000	0.000000	0.000000	0.000000
10	0.000000	0.000000	0.000000	0.000000	0.000000	0.000000
11	0.000000	0.000000	0.000000	0.000000	0.000000	0.000000
12	0.059113	0.000000	0.000000	0.000000	0.000000	0.000000
13	0.059113	0.000000	0.000000	0.000000	0.000000	0.000000
14	0.000000	0.000000	0.000000	0.000000	0.000000	0.000000
15	0.000000	0.000000	0.000000	0.000000	0.000000	0.000000
16	0.000000	0.000000	0.000000	0.000000	0.000000	0.000000
17	0.000000	0.000000	0.000000	0.000000	0.000000	0.000000
18	0.000000	0.000000	0.000000	0.000000	0.000000	0.000000
19	0.070857	0.000000	0.036115	0.042671	0.027696	0.000000
20	0.061067	0.000000	0.047406	0.030553	0.038313	0.000000
21	0.051482	0.000000	0.044213	0.031603	0.050042	0.000000
22	0.044205	0.000000	0.043672	0.044030	0.045433	0.000000
23	0.043302	0.000000	0.055104	0.027856	0.051078	0.000000
24	0.042822	0.000000	0.043448	0.037693	0.053376	0.000000
25	0.000000	0.000000	0.006329	0.004445	0.012224	0.001936
26	0.000000	0.000000	0.008076	0.004638	0.008150	0.001757
27	0.000000	0.000000	0.005714	0.004038	0.012423	0.001415
28	0.000000	0.000000	0.006067	0.003680	0.006687	0.001088
29	0.000000	0.000000	0.000000	0.000000	0.000000	0.000000
30	0.000000	0.354680	0.000000	0.000000	0.000000	0.000000
31	0.000000	0.000019	0.000000	0.000000	0.000000	0.000000
32	0.000000	0.354661	0.000000	0.000000	0.000000	0.000000
33	0.000000	0.000000	0.103963	0.000000	0.000000	0.000000
34	0.000000	0.000000	0.136154	0.000000	0.000000	0.000000
35	0.000000	0.000000	0.114563	0.000000	0.000000	0.000000
36	0.000000	0.000000	0.000000	0.115408	0.000000	0.000000
37	0.000000	0.000000	0.000000	0.119058	0.000000	0.000000
38	0.000000	0.000000	0.000000	0.120214	0.000000	0.000000
39	0.000000	0.000000	0.000000	0.000000	0.114795	0.000000
40	0.000000	0.000000	0.000000	0.000000	0.131735	0.000000
41	0.000000	0.000000	0.000000	0.000000	0.108150	0.000000
42	0.000000	0.000000	0.000000	0.000000	0.000000	0.116794
43	0.000000	0.000000	0.000000	0.000000	0.000000	0.116375
44	0.000000	0.000000	0.000000	0.000000	0.000000	0.121511
45	0.039426	0.001110	0.006938	0.009151	0.016719	0.015326

Legends: Act – Activities; Subpop – Subpopulation;
Columns – Frequencies on all activities for each subpopulation.

S4 Table 7 (Continue): Distancing Strategies at Equilibrium from Test 6

Act	Subpop 1	Subpop 2	Subpop 3	Subpop 4	Subpop 5	Subpop 6
46	0.000000	0.000000	0.000000	0.000000	0.000000	0.000000
47	0.000000	0.000000	0.000000	0.000000	0.000000	0.000000
48	0.000000	0.000000	0.000000	0.000000	0.000000	0.000000
49	0.097051	0.000000	0.013842	0.023873	0.034154	0.008420
50	0.099805	0.000002	0.014053	0.023936	0.029295	0.010181
51	0.000000	0.000000	0.000000	0.000000	0.000000	0.000000
52	0.019893	0.000000	0.002503	0.006787	0.010551	0.002401
53	0.000000	0.000000	0.000000	0.000000	0.000000	0.000000
54	0.016588	0.000000	0.002890	0.010724	0.011906	0.004427
55	0.000000	0.000000	0.000000	0.000000	0.000000	0.000000
56	0.000038	0.000003	0.000002	0.000006	0.000019	0.000000
57	0.000000	0.065039	0.000000	0.000000	0.000000	0.112301
58	0.000000	0.000000	0.000000	0.000000	0.000000	0.000000
59	0.000000	0.080732	0.000000	0.000000	0.000000	0.096608
60	0.000000	0.000000	0.000000	0.000000	0.000000	0.000000
61	0.000000	0.076860	0.000000	0.000000	0.000000	0.100480
62	0.000000	0.000000	0.000000	0.000000	0.000000	0.000000
63	0.000000	0.049743	0.000000	0.000000	0.000000	0.127597
64	0.000000	0.000000	0.000000	0.000000	0.000000	0.000045
65	0.000000	0.000000	0.000000	0.000000	0.000000	0.000000
66	0.000000	0.004781	0.000000	0.000000	0.000000	0.035625
67	0.000000	0.003823	0.000000	0.000000	0.000000	0.044395
68	0.000000	0.000000	0.000000	0.000000	0.000000	0.000000
69	0.000000	0.000000	0.027843	0.038625	0.014176	0.008026
70	0.000000	0.000000	0.000000	0.000000	0.000000	0.000000
71	0.000000	0.000001	0.028297	0.038798	0.012058	0.009516
72	0.000000	0.000000	0.000000	0.000000	0.000000	0.000000
73	0.000000	0.000000	0.000000	0.000000	0.000000	0.000000
74	0.000000	0.000000	0.023375	0.036254	0.020879	0.008162
75	0.000000	0.000000	0.000000	0.000000	0.000000	0.000000
76	0.000000	0.005028	0.039212	0.059536	0.044948	0.028616
77	0.000000	0.000000	0.000000	0.000000	0.000000	0.000000
78	0.000000	0.000000	0.000000	0.000000	0.000000	0.000000
79	0.000000	0.003493	0.059152	0.049252	0.054596	0.010847
80	0.000000	0.000017	0.038094	0.022055	0.025542	0.002962
81	0.000000	0.000006	0.032133	0.023785	0.025942	0.006804
82	0.000000	0.000000	0.028613	0.041343	0.015895	0.002818
83	0.000000	0.000000	0.000000	0.000000	0.000000	0.000000
84	0.000000	0.000000	0.000000	0.000000	0.000000	0.000000
85	0.000000	0.000000	0.032049	0.029982	0.023091	0.003547
86						
87						
88						
89						
90						

Legends: Act – Activities; Subpop – Subpopulation;
Columns – Frequencies on all activities for each subpopulation.

S4 Table 8: Distancing Strategies at Equilibrium from Test 7

Act	Subpop 1	Subpop 2	Subpop 3	Subpop 4	Subpop 5	Subpop 6
1	0.000000	0.000000	0.000001	0.000000	0.000004	0.000000
2	0.000000	0.000000	0.000000	0.000000	0.000000	0.000000
3	0.000000	0.000000	0.000000	0.000000	0.000000	0.000000
4	0.000000	0.000000	0.000000	0.000000	0.000000	0.000000
5	0.059064	0.000000	0.000007	0.000001	0.000012	0.000022
6	0.053856	0.000000	0.000364	0.000003	0.000667	0.004224
7	0.058798	0.000000	0.000004	0.000000	0.000000	0.000311
8	0.059101	0.000000	0.000005	0.000000	0.000002	0.000004
9	0.000000	0.000000	0.000000	0.000000	0.000000	0.000000
10	0.059113	0.000000	0.000000	0.000000	0.000000	0.000000
11	0.059113	0.000000	0.000000	0.000000	0.000000	0.000000
12	0.000000	0.000000	0.000000	0.000000	0.000000	0.000000
13	0.000000	0.000000	0.000000	0.000000	0.000000	0.000000
14	0.059113	0.000000	0.000000	0.000000	0.000000	0.000000
15	0.000003	0.000000	0.000001	0.000002	0.000046	0.000000
16	0.000000	0.000000	0.000000	0.000000	0.000000	0.000000
17	0.000000	0.000000	0.000000	0.000000	0.000000	0.000000
18	0.000000	0.000000	0.000000	0.000000	0.000000	0.000000
19	0.054867	0.000000	0.030769	0.047332	0.044320	0.000000
20	0.061248	0.000000	0.031503	0.049084	0.035504	0.000000
21	0.048136	0.000000	0.050364	0.035561	0.043279	0.000000
22	0.052490	0.000000	0.049935	0.034514	0.040401	0.000000
23	0.049852	0.000000	0.044802	0.044306	0.038379	0.000000
24	0.061172	0.000000	0.043025	0.036294	0.036849	0.000000
25	0.000000	0.000000	0.006582	0.004368	0.012533	0.001846
26	0.000000	0.000000	0.005253	0.002500	0.010421	0.001187
27	0.000000	0.000000	0.006542	0.003002	0.011781	0.001574
28	0.000000	0.000000	0.006702	0.002705	0.009581	0.002094
29	0.000000	0.000000	0.000000	0.000000	0.000000	0.000000
30	0.000000	0.354680	0.000000	0.000000	0.000000	0.000000
31	0.000000	0.000001	0.000000	0.000000	0.000000	0.000000
32	0.000000	0.354679	0.000000	0.000000	0.000000	0.000000
33	0.000000	0.000000	0.112106	0.000000	0.000000	0.000000
34	0.000000	0.000000	0.128014	0.000000	0.000000	0.000000
35	0.000000	0.000000	0.114559	0.000000	0.000000	0.000000
36	0.000000	0.000000	0.000000	0.112895	0.000000	0.000000
37	0.000000	0.000000	0.000000	0.121069	0.000000	0.000000
38	0.000000	0.000000	0.000000	0.120715	0.000000	0.000000
39	0.000000	0.000000	0.000000	0.000000	0.128540	0.000000
40	0.000000	0.000000	0.000000	0.000000	0.123150	0.000000
41	0.000000	0.000000	0.000000	0.000000	0.102990	0.000000
42	0.000000	0.000000	0.000000	0.000000	0.000000	0.131573
43	0.000000	0.000000	0.000000	0.000000	0.000000	0.111958
44	0.000000	0.000000	0.000000	0.000000	0.000000	0.111149
45	0.021681	0.000828	0.009371	0.007719	0.045277	0.003794

Legends: Act – Activities; Subpop – Subpopulation;
Columns – Frequencies on all activities for each subpopulation.

S4 Table 8 (Continue): Distancing Strategies at Equilibrium from Test 7

Act	Subpop 1	Subpop 2	Subpop 3	Subpop 4	Subpop 5	Subpop 6
46	0.000000	0.000000	0.000000	0.000000	0.000000	0.000000
47	0.000000	0.000000	0.000000	0.000000	0.000000	0.000000
48	0.000000	0.000000	0.000000	0.000000	0.000000	0.000000
49	0.105814	0.000002	0.008235	0.029074	0.030763	0.003453
50	0.100446	0.000001	0.009756	0.027401	0.030064	0.009210
51	0.000000	0.000000	0.000000	0.000000	0.000000	0.000000
52	0.015003	0.000000	0.002812	0.010667	0.003291	0.008691
53	0.000000	0.000000	0.000000	0.000000	0.000000	0.000000
54	0.020928	0.000000	0.001934	0.010647	0.003098	0.011599
55	0.000000	0.000000	0.000000	0.000000	0.000000	0.000000
56	0.000199	0.000001	0.000029	0.000074	0.000155	0.000003
57	0.000000	0.050530	0.000000	0.000000	0.000000	0.126810
58	0.000000	0.000000	0.000000	0.000000	0.000000	0.000000
59	0.000000	0.087435	0.000000	0.000000	0.000000	0.089905
60	0.000000	0.000000	0.000000	0.000000	0.000000	0.000000
61	0.000000	0.080309	0.000000	0.000000	0.000000	0.097031
62	0.000000	0.000000	0.000000	0.000000	0.000000	0.000000
63	0.000000	0.051630	0.000000	0.000000	0.000000	0.125710
64	0.000000	0.000001	0.000000	0.000000	0.000000	0.000005
65	0.000000	0.000000	0.000000	0.000000	0.000000	0.000000
66	0.000000	0.002704	0.000000	0.000000	0.000000	0.040837
67	0.000000	0.005535	0.000000	0.000000	0.000000	0.039589
68	0.000000	0.000000	0.000000	0.000000	0.000000	0.000000
69	0.000000	0.000000	0.030867	0.034187	0.016701	0.006915
70	0.000000	0.000000	0.000000	0.000000	0.000000	0.000000
71	0.000000	0.000001	0.029046	0.034268	0.018329	0.007026
72	0.000000	0.000000	0.000000	0.000000	0.000000	0.000000
73	0.000000	0.000000	0.000000	0.000000	0.000000	0.000000
74	0.000000	0.000001	0.027202	0.022922	0.020350	0.018195
75	0.000000	0.000000	0.000000	0.000000	0.000000	0.000000
76	0.000000	0.000000	0.000000	0.000000	0.000000	0.000000
77	0.000000	0.004662	0.053667	0.042168	0.066268	0.010574
78	0.000000	0.006869	0.074312	0.034979	0.051060	0.010119
79	0.000000	0.000000	0.000000	0.000000	0.000000	0.000000
80	0.000000	0.000076	0.024500	0.036187	0.022043	0.005865
81	0.000000	0.000057	0.028824	0.031671	0.019346	0.008772
82	0.000000	0.000001	0.036016	0.032126	0.016673	0.003854
83	0.000000	0.000000	0.000000	0.000000	0.000000	0.000000
84	0.000000	0.000000	0.000000	0.000000	0.000000	0.000000
85	0.000000	0.000000	0.032890	0.031555	0.018124	0.006101
86						
87						
88						
89						
90						

Legends: Act – Activities; Subpop – Subpopulation;
Columns – Frequencies on all activities for each subpopulation.

S4 Table 9: Distancing Strategies at Equilibrium from Test 8

Act	Subpop 1	Subpop 2	Subpop 3	Subpop 4	Subpop 5	Subpop 6
1	0.000000	0.000000	0.000000	0.000000	0.000000	0.000000
2	0.000000	0.000000	0.000000	0.000000	0.000000	0.000000
3	0.000000	0.000000	0.000000	0.000000	0.000000	0.000000
4	0.000000	0.000000	0.000000	0.000000	0.000000	0.000000
5	0.059067	0.000000	0.000006	0.000001	0.000003	0.000036
6	0.059017	0.000000	0.000016	0.000001	0.000002	0.000077
7	0.051319	0.000000	0.000035	0.000003	0.000957	0.006799
8	0.051301	0.000000	0.002938	0.000007	0.002421	0.002446
9	0.000000	0.000000	0.000000	0.000000	0.000000	0.000000
10	0.059113	0.000000	0.000000	0.000000	0.000000	0.000000
11	0.059113	0.000000	0.000000	0.000000	0.000000	0.000000
12	0.000000	0.000000	0.000000	0.000000	0.000000	0.000000
13	0.000000	0.000000	0.000000	0.000000	0.000000	0.000000
14	0.059113	0.000000	0.000000	0.000000	0.000000	0.000000
15	0.000000	0.000000	0.000000	0.000000	0.000000	0.000000
16	0.000027	0.000000	0.000001	0.000002	0.000013	0.000000
17	0.000000	0.000000	0.000000	0.000000	0.000000	0.000000
18	0.000000	0.000000	0.000000	0.000000	0.000000	0.000000
19	0.075540	0.000000	0.028540	0.033988	0.039272	0.000000
20	0.070195	0.000000	0.030297	0.039038	0.037766	0.000000
21	0.045779	0.000000	0.036604	0.046221	0.048736	0.000000
22	0.031416	0.000000	0.059026	0.039424	0.047475	0.000000
23	0.023282	0.000000	0.054175	0.040590	0.059293	0.000000
24	0.037149	0.000000	0.051972	0.041497	0.046722	0.000000
25	0.000000	0.000000	0.008096	0.001339	0.007034	0.003035
26	0.000000	0.000000	0.006989	0.001612	0.009383	0.002042
27	0.000000	0.000000	0.014298	0.002476	0.008923	0.002152
28	0.000000	0.000000	0.007283	0.002009	0.008158	0.003843
29	0.000000	0.000153	0.000000	0.000000	0.000000	0.000000
30	0.000000	0.354526	0.000000	0.000000	0.000000	0.000000
31	0.000000	0.000000	0.000000	0.000000	0.000000	0.000000
32	0.000000	0.354680	0.000000	0.000000	0.000000	0.000000
33	0.000000	0.000000	0.116743	0.000000	0.000000	0.000000
34	0.000000	0.000000	0.112673	0.000000	0.000000	0.000000
35	0.000000	0.000000	0.125264	0.000000	0.000000	0.000000
36	0.000000	0.000000	0.000000	0.135443	0.000000	0.000000
37	0.000000	0.000000	0.000000	0.115730	0.000000	0.000000
38	0.000000	0.000000	0.000000	0.103507	0.000000	0.000000
39	0.000000	0.000000	0.000000	0.000000	0.115933	0.000000
40	0.000000	0.000000	0.000000	0.000000	0.114700	0.000000
41	0.000000	0.000000	0.000000	0.000000	0.124047	0.000000
42	0.000000	0.000000	0.000000	0.000000	0.000000	0.123993
43	0.000000	0.000000	0.000000	0.000000	0.000000	0.114401
44	0.000000	0.000000	0.000000	0.000000	0.000000	0.116286
45	0.084377	0.000615	0.001344	0.000591	0.000605	0.001138

Legends: Act – Activities; Subpop – Subpopulation;
Columns – Frequencies on all activities for each subpopulation.

S4 Table 9 (Continue): Distancing Strategies at Equilibrium from Test 8

Act	Subpop 1	Subpop 2	Subpop 3	Subpop 4	Subpop 5	Subpop 6
46	0.000000	0.000000	0.000000	0.000000	0.000000	0.000000
47	0.000000	0.000000	0.000000	0.000000	0.000000	0.000000
48	0.000000	0.000000	0.000000	0.000000	0.000000	0.000000
49	0.076798	0.000019	0.032630	0.026484	0.030574	0.010834
50	0.082313	0.000021	0.028991	0.028417	0.031211	0.006385
51	0.000000	0.000000	0.000000	0.000000	0.000000	0.000000
52	0.039089	0.000000	0.002315	0.001220	0.001007	0.001046
53	0.000000	0.000000	0.000000	0.000000	0.000000	0.000000
54	0.035991	0.000000	0.001545	0.002965	0.000725	0.002767
55	0.000000	0.000002	0.000000	0.000000	0.000000	0.000000
56	0.000000	0.000001	0.000000	0.000000	0.000000	0.000000
57	0.000000	0.048213	0.000000	0.000000	0.000000	0.129127
58	0.000000	0.000000	0.000000	0.000000	0.000000	0.000000
59	0.000000	0.073421	0.000000	0.000000	0.000000	0.103919
60	0.000000	0.000000	0.000000	0.000000	0.000000	0.000000
61	0.000000	0.107656	0.000000	0.000000	0.000000	0.069683
62	0.000000	0.000000	0.000000	0.000000	0.000000	0.000000
63	0.000000	0.042786	0.000000	0.000000	0.000000	0.134554
64	0.000000	0.000000	0.000000	0.000000	0.000000	0.000005
65	0.000000	0.000000	0.000000	0.000000	0.000000	0.000000
66	0.000000	0.001840	0.000000	0.000000	0.000000	0.043708
67	0.000000	0.001805	0.000000	0.000000	0.000000	0.041312
68	0.000000	0.000000	0.000000	0.000000	0.000000	0.000000
69	0.000000	0.000000	0.028185	0.032291	0.017495	0.010698
70	0.000000	0.000000	0.000000	0.000000	0.000000	0.000000
71	0.000000	0.000000	0.029877	0.037826	0.015781	0.005185
72	0.000000	0.000000	0.000000	0.000000	0.000000	0.000000
73	0.000000	0.000000	0.000000	0.000000	0.000000	0.000000
74	0.000000	0.000000	0.029099	0.031430	0.016931	0.011209
75	0.000000	0.000000	0.000000	0.000000	0.000000	0.000000
76	0.000000	0.005584	0.047751	0.032170	0.071794	0.020040
77	0.000000	0.000000	0.000000	0.000000	0.000000	0.000000
78	0.000000	0.000000	0.000000	0.000000	0.000000	0.000000
79	0.000000	0.008540	0.050910	0.030883	0.072017	0.014990
80	0.000000	0.000134	0.022777	0.038467	0.019924	0.007367
81	0.000000	0.000001	0.023166	0.038743	0.018631	0.008129
82	0.000000	0.000000	0.027284	0.047928	0.011557	0.001900
83	0.000000	0.000000	0.000000	0.000000	0.000000	0.000000
84	0.000000	0.000000	0.000000	0.000000	0.000000	0.000000
85	0.000000	0.000000	0.019169	0.047698	0.020910	0.000892
86						
87						
88						
89						
90						

Legends: Act – Activities; Subpop – Subpopulation;
Columns – Frequencies on all activities for each subpopulation.

S4 Table 10: Distancing Strategies at Equilibrium from Test 9

Act	Subpop 1	Subpop 2	Subpop 3	Subpop 4	Subpop 5	Subpop 6
1	0.000000	0.000000	0.000000	0.000000	0.000000	0.000000
2	0.000011	0.000000	0.000000	0.000000	0.000000	0.000000
3	0.000000	0.000000	0.000000	0.000000	0.000000	0.000000
4	0.000000	0.000000	0.000000	0.000000	0.000000	0.000000
5	0.056316	0.000000	0.000248	0.000000	0.000351	0.002199
6	0.058677	0.000000	0.000008	0.000000	0.000000	0.000417
7	0.059095	0.000000	0.000009	0.000000	0.000001	0.000008
8	0.058488	0.000000	0.000035	0.000007	0.000025	0.000558
9	0.000000	0.000000	0.000000	0.000000	0.000000	0.000000
10	0.059113	0.000000	0.000000	0.000000	0.000000	0.000000
11	0.059113	0.000000	0.000000	0.000000	0.000000	0.000000
12	0.000000	0.000000	0.000000	0.000000	0.000000	0.000000
13	0.000000	0.000000	0.000000	0.000000	0.000000	0.000000
14	0.059113	0.000000	0.000000	0.000000	0.000000	0.000000
15	0.000000	0.000000	0.000000	0.000000	0.000000	0.000000
16	0.000003	0.000000	0.000001	0.000009	0.000028	0.000000
17	0.000000	0.000000	0.000000	0.000000	0.000000	0.000000
18	0.000000	0.000000	0.000000	0.000000	0.000000	0.000000
19	0.070084	0.000000	0.033068	0.038398	0.035790	0.000000
20	0.070777	0.000000	0.033184	0.038693	0.034645	0.000000
21	0.046380	0.000000	0.050815	0.024377	0.055767	0.000000
22	0.047489	0.000000	0.049807	0.026890	0.053154	0.000000
23	0.038879	0.000000	0.050205	0.034728	0.053527	0.000000
24	0.044436	0.000000	0.052262	0.025905	0.054737	0.000000
25	0.000000	0.000005	0.011564	0.001303	0.007421	0.004128
26	0.000000	0.000003	0.010304	0.002520	0.006329	0.001739
27	0.000000	0.000002	0.010727	0.002368	0.009477	0.001499
28	0.000000	0.000005	0.007896	0.002089	0.006242	0.003049
29	0.000000	0.000000	0.000000	0.000000	0.000000	0.000000
30	0.000000	0.354680	0.000000	0.000000	0.000000	0.000000
31	0.000000	0.000013	0.000000	0.000000	0.000000	0.000000
32	0.000000	0.354667	0.000000	0.000000	0.000000	0.000000
33	0.000000	0.000000	0.110155	0.000000	0.000000	0.000000
34	0.000000	0.000000	0.103325	0.000000	0.000000	0.000000
35	0.000000	0.000000	0.141200	0.000000	0.000000	0.000000
36	0.000000	0.000000	0.000000	0.117511	0.000000	0.000000
37	0.000000	0.000000	0.000000	0.104510	0.000000	0.000000
38	0.000000	0.000000	0.000000	0.132658	0.000000	0.000000
39	0.000000	0.000000	0.000000	0.000000	0.114314	0.000000
40	0.000000	0.000000	0.000000	0.000000	0.122743	0.000000
41	0.000000	0.000000	0.000000	0.000000	0.117623	0.000000
42	0.000000	0.000000	0.000000	0.000000	0.000000	0.125549
43	0.000000	0.000000	0.000000	0.000000	0.000000	0.114083
44	0.000000	0.000000	0.000000	0.000000	0.000000	0.115048
45	0.043243	0.000766	0.004490	0.006078	0.029764	0.004329

Legends: Act – Activities; Subpop – Subpopulation;
Columns – Frequencies on all activities for each subpopulation.

S4 Table 10 (Continue): Distancing Strategies at Equilibrium from Test 9

Act	Subpop 1	Subpop 2	Subpop 3	Subpop 4	Subpop 5	Subpop 6
46	0.000000	0.000000	0.000000	0.000000	0.000000	0.000000
47	0.000000	0.000000	0.000000	0.000000	0.000000	0.000000
48	0.000000	0.000000	0.000000	0.000000	0.000000	0.000000
49	0.097302	0.000013	0.016668	0.026183	0.023942	0.013233
50	0.098993	0.000004	0.010803	0.031887	0.020267	0.015381
51	0.000000	0.000000	0.000000	0.000000	0.000000	0.000000
52	0.016920	0.000000	0.003442	0.014819	0.004964	0.005672
53	0.000000	0.000000	0.000000	0.000000	0.000000	0.000000
54	0.015565	0.000000	0.003794	0.013164	0.005899	0.004432
55	0.000000	0.000000	0.000000	0.000000	0.000000	0.000000
56	0.000003	0.000001	0.000001	0.000000	0.000001	0.000000
57	0.000000	0.049279	0.000000	0.000000	0.000000	0.128061
58	0.000000	0.000000	0.000000	0.000000	0.000000	0.000000
59	0.000000	0.082797	0.000000	0.000000	0.000000	0.094543
60	0.000000	0.000000	0.000000	0.000000	0.000000	0.000000
61	0.000000	0.079226	0.000000	0.000000	0.000000	0.098114
62	0.000000	0.000000	0.000000	0.000000	0.000000	0.000000
63	0.000000	0.068275	0.000000	0.000000	0.000000	0.109065
64	0.000000	0.000000	0.000000	0.000000	0.000000	0.000001
65	0.000000	0.000000	0.000000	0.000000	0.000000	0.000000
66	0.000000	0.000743	0.000000	0.000000	0.000000	0.040679
67	0.000000	0.001206	0.000000	0.000000	0.000000	0.046041
68	0.000000	0.000000	0.000000	0.000000	0.000000	0.000000
69	0.000000	0.000000	0.029111	0.038830	0.012743	0.007986
70	0.000000	0.000000	0.000000	0.000000	0.000000	0.000000
71	0.000000	0.000000	0.023504	0.036313	0.021633	0.007220
72	0.000000	0.000000	0.000000	0.000000	0.000000	0.000000
73	0.000000	0.000000	0.000000	0.000000	0.000000	0.000000
74	0.000000	0.000000	0.032167	0.026147	0.020766	0.009590
75	0.000000	0.000000	0.000000	0.000000	0.000000	0.000000
76	0.000000	0.000000	0.000000	0.000000	0.000000	0.000000
77	0.000000	0.003981	0.042602	0.045612	0.077668	0.007476
78	0.000000	0.004174	0.056505	0.053171	0.053496	0.009993
79	0.000000	0.000000	0.000000	0.000000	0.000000	0.000000
80	0.000000	0.000092	0.026564	0.039821	0.012184	0.010009
81	0.000000	0.000069	0.025656	0.039764	0.011890	0.011292
82	0.000000	0.000000	0.027634	0.037891	0.017589	0.005556
83	0.000000	0.000000	0.000000	0.000000	0.000000	0.000000
84	0.000000	0.000000	0.000000	0.000000	0.000000	0.000000
85	0.000000	0.000000	0.032244	0.038353	0.015020	0.003052
86						
87						
88						
89						
90						

Legends: Act – Activities; Subpop – Subpopulation;
Columns – Frequencies on all activities for each subpopulation.

S4 Table 11: Distancing Strategies at Equilibrium from Test 10

Act	Subpop 1	Subpop 2	Subpop 3	Subpop 4	Subpop 5	Subpop 6
1	0.000000	0.000000	0.000000	0.000000	0.000000	0.000000
2	0.000000	0.000000	0.000000	0.000000	0.000000	0.000000
3	0.000000	0.000000	0.000000	0.000000	0.000000	0.000000
4	0.000000	0.000000	0.000000	0.000000	0.000000	0.000000
5	0.059100	0.000000	0.000001	0.000000	0.000000	0.000012
6	0.059048	0.000000	0.000015	0.000024	0.000026	0.000000
7	0.058428	0.000000	0.000005	0.000006	0.000085	0.000589
8	0.055719	0.000000	0.000023	0.000001	0.000587	0.002784
9	0.059113	0.000000	0.000000	0.000000	0.000000	0.000000
10	0.000000	0.000000	0.000000	0.000000	0.000000	0.000000
11	0.000000	0.000000	0.000000	0.000000	0.000000	0.000000
12	0.059113	0.000000	0.000000	0.000000	0.000000	0.000000
13	0.059113	0.000000	0.000000	0.000000	0.000000	0.000000
14	0.000000	0.000000	0.000000	0.000000	0.000000	0.000000
15	0.000004	0.000000	0.000001	0.000005	0.000014	0.000000
16	0.000000	0.000000	0.000000	0.000000	0.000000	0.000000
17	0.000000	0.000000	0.000000	0.000000	0.000000	0.000000
18	0.000000	0.000000	0.000000	0.000000	0.000000	0.000000
19	0.057717	0.000000	0.039266	0.040599	0.039734	0.000000
20	0.062359	0.000000	0.030720	0.048839	0.035421	0.000000
21	0.033737	0.000000	0.055700	0.035729	0.052174	0.000000
22	0.040012	0.000000	0.053277	0.033877	0.050174	0.000000
23	0.034121	0.000000	0.055387	0.033946	0.053886	0.000000
24	0.051117	0.000000	0.033823	0.042114	0.050285	0.000000
25	0.000000	0.000000	0.008378	0.005040	0.002991	0.005817
26	0.000000	0.000000	0.006445	0.004355	0.004237	0.007728
27	0.000000	0.000001	0.008550	0.004049	0.003435	0.005194
28	0.000000	0.000000	0.009061	0.005060	0.002572	0.005760
29	0.000000	0.000000	0.000000	0.000000	0.000000	0.000000
30	0.000000	0.354680	0.000000	0.000000	0.000000	0.000000
31	0.000000	0.000000	0.000000	0.000000	0.000000	0.000000
32	0.000000	0.354680	0.000000	0.000000	0.000000	0.000000
33	0.000000	0.000000	0.111000	0.000000	0.000000	0.000000
34	0.000000	0.000000	0.115369	0.000000	0.000000	0.000000
35	0.000000	0.000000	0.128311	0.000000	0.000000	0.000000
36	0.000000	0.000000	0.000000	0.126341	0.000000	0.000000
37	0.000000	0.000000	0.000000	0.099021	0.000000	0.000000
38	0.000000	0.000000	0.000000	0.129318	0.000000	0.000000
39	0.000000	0.000000	0.000000	0.000000	0.126667	0.000000
40	0.000000	0.000000	0.000000	0.000000	0.109465	0.000000
41	0.000000	0.000000	0.000000	0.000000	0.118548	0.000000
42	0.000000	0.000000	0.000000	0.000000	0.000000	0.123663
43	0.000000	0.000000	0.000000	0.000000	0.000000	0.129224
44	0.000000	0.000000	0.000000	0.000000	0.000000	0.101793
45	0.007115	0.000000	0.000521	0.037427	0.041127	0.002479

Legends: Act – Activities; Subpop – Subpopulation;
Columns – Frequencies on all activities for each subpopulation.

S4 Table 11 (Continue): Distancing Strategies at Equilibrium from Test 10

Act	Subpop 1	Subpop 2	Subpop 3	Subpop 4	Subpop 5	Subpop 6
46	0.000000	0.000000	0.000000	0.000000	0.000000	0.000000
47	0.000000	0.000000	0.000000	0.000000	0.000000	0.000000
48	0.000000	0.000000	0.000000	0.000000	0.000000	0.000000
49	0.123180	0.000004	0.000324	0.018956	0.030959	0.003662
50	0.101464	0.000003	0.009153	0.033822	0.029278	0.003620
51	0.000000	0.000000	0.000000	0.000000	0.000000	0.000000
52	0.044385	0.000000	0.001927	0.001133	0.001767	0.000650
53	0.000000	0.000000	0.000000	0.000000	0.000000	0.000000
54	0.035122	0.000000	0.001782	0.000148	0.000484	0.001272
55	0.000032	0.000001	0.000000	0.000058	0.000162	0.000002
56	0.000000	0.000000	0.000000	0.000000	0.000000	0.000000
57	0.000000	0.047520	0.000000	0.000000	0.000000	0.129820
58	0.000000	0.000000	0.000000	0.000000	0.000000	0.000000
59	0.000000	0.090062	0.000000	0.000000	0.000000	0.087277
60	0.000000	0.000000	0.000000	0.000000	0.000000	0.000000
61	0.000000	0.091045	0.000000	0.000000	0.000000	0.086295
62	0.000000	0.000000	0.000000	0.000000	0.000000	0.000000
63	0.000000	0.040502	0.000000	0.000000	0.000000	0.136838
64	0.000000	0.000000	0.000000	0.000000	0.000000	0.000002
65	0.000000	0.000000	0.000000	0.000000	0.000000	0.000000
66	0.000000	0.001753	0.000000	0.000000	0.000000	0.043053
67	0.000000	0.001601	0.000000	0.000000	0.000000	0.042261
68	0.000000	0.000000	0.000000	0.000000	0.000000	0.000000
69	0.000000	0.000004	0.038864	0.032142	0.015473	0.002186
70	0.000000	0.000000	0.000000	0.000000	0.000000	0.000000
71	0.000000	0.000002	0.025669	0.033666	0.021734	0.007599
72	0.000000	0.000000	0.000000	0.000000	0.000000	0.000000
73	0.000000	0.000000	0.000000	0.000002	0.000000	0.000000
74	0.000000	0.000033	0.025077	0.036035	0.020234	0.007290
75	0.000000	0.000000	0.000000	0.000000	0.000000	0.000000
76	0.000000	0.009822	0.054619	0.026509	0.057012	0.029378
77	0.000000	0.000000	0.000000	0.000000	0.000000	0.000000
78	0.000000	0.000000	0.000000	0.000000	0.000000	0.000000
79	0.000000	0.008109	0.066206	0.043621	0.048857	0.010547
80	0.000000	0.000045	0.034078	0.026463	0.021490	0.006593
81	0.000000	0.000060	0.031449	0.031568	0.018265	0.007327
82	0.000000	0.000000	0.030597	0.032609	0.020729	0.004736
83	0.000000	0.000000	0.000000	0.000000	0.000000	0.000000
84	0.000000	0.000000	0.000000	0.000000	0.000000	0.000000
85	0.000000	0.000072	0.024401	0.037518	0.022128	0.004550
86						
87						
88						
89						
90						

Legends: Act – Activities; Subpop – Subpopulation;
Columns – Frequencies on all activities for each subpopulation.
