# Supplementary material for: Social distancing is a social dilemma game played by every individual against his/her population
Source: PLoS One. 2021 Aug 2;16(8):e0255543. doi: 10.1371/journal.pone.0255543 (PMC8328347; doi:10.1371/journal.pone.0255543)
Supplement: S5 File — Code for simulation for a small single population with 10 social activities. (PDF) [file pone.0255543.s005.pdf]

S5 File. Simulation Code 1: Matlab code for simulation on small single populations.

README:

Simulation for the population in Fig 2:

A1.dat, w1.dat -- connectivity matrix and weights when activities are dependent.

A2.dat, w2.dat -- connectivity matrix and weights when activities are independent.

Copy them to A.dat and w.dat

Simulation for a population that forms a Petersen's diagram:

A3.dat, w3.dat -- connectivity matrix and weights.

Copy them to A.dat and w.dat

In Matlab, type:

>test_run_1

There are a lot of pauses in the code so you can look the intermediate results and figures. Hit any key once or twice to continue.

Ending frequencies are saved in pop_stg_eq_*.dat.

A1.dat:

```
1 1 1 1 0 1 0 0 0 0
1 1 1 1 0 0 0 0 1 0
1 1 1 1 0 1 0 0 0 0
1 1 1 1 0 0 0 0 1 0
0 0 0 0 1 1 1 0 0 0
1 0 1 0 1 1 1 0 0 0
0 0 0 0 1 1 1 0 0 0
0 0 0 0 0 0 0 1 1 1
0 1 0 1 0 0 0 1 1 1
0 0 0 0 0 0 0 1 1 1
```

w1.dat:

```
4 4 4 4 1 1 1 1 1 1
```

A2.dat:

```
1 0 0 0 0 0 0 0 0 0
0 1 0 0 0 0 0 0 0 0
0 0 1 0 0 0 0 0 0 0
0 0 0 1 0 0 0 0 0 0
0 0 0 0 1 0 0 0 0 0
0 0 0 0 0 1 0 0 0 0
0 0 0 0 0 0 1 0 0 0
0 0 0 0 0 0 0 1 0 0
0 0 0 0 0 0 0 0 1 0
0 0 0 0 0 0 0 0 0 1
```

w2.dat:

```
4 4 4 4 1 1 1 1 1 1
```

A3.dat:

```
1 1 0 0 1 1 0 0 0 0
1 1 1 0 0 0 1 0 0 0
0 1 1 1 0 0 0 1 0 0
0 0 1 1 1 0 0 0 1 0
1 0 0 1 1 0 0 0 0 1
1 0 0 0 0 1 0 1 1 0
0 1 0 0 0 0 1 0 1 1
0 0 1 0 0 1 0 1 0 1
0 0 0 1 0 1 1 0 1 0
0 0 0 0 1 0 1 1 0 1
```

w3.dat:

```
4 4 4 4 4 1 1 1 1 1
```

test_run_1.m:

```
function [ret_info,ind_stg_eq,pop_stg_eq] = test_run_1 ()

%
% Simulation of collective behavior of social distancing
%
% (Small, single population)
%
% m -- # individuals in population, m = 100
%
% A -- connectivity matrix of social activities, n x n, n = 10
% w -- contact weights assigned to social activities, n x 1
%
% ind_stg_in -- initial strategies of individuals, n x m
% pop_stg_in -- initial strategy of population, n x 1
%
% ind_stg_eq -- equilibrium strategies of individuals, n x m
% pop_stg_eq -- equilibrium strategy of population, n x 1
%
% ret_info -- 1 -- suceeds, 0 -- fails
%
% Zhijun Wu, 12/20/2020, Math Dept, Iowa State University
%

% Load contact matrix and contact weights

A = load('A.dat','-ascii');
w = load('w.dat','-ascii');

k = size(A,1);

% Obtain # strategies and # individuals

n = k;
m = 10*n;

K = 100;

% Start with initial random strategies

rng ('default');

% ind_stg_in = rand(n,m);

for i = 0 : n-1          % Repeat with different initials

   for j = 0 : n-1
```

```
        k = rem(i+j,n)+1;
        ind_stg_in(k,1:m) = (j+1)*rand(1,m);
    end

    for l = 1 : m
        ind_stg_in(1:n,l) = ind_stg_in(1:n,l) / sum(ind_stg_in(1:n,l));
    end
    pop_stg_in = sum(ind_stg_in,2) / m;

    % Start simulation, to reach equilibrium strategies

    [ind_stg_eq,pop_stg_eq] = soc_dis_sim (ind_stg_in,pop_stg_in,A,w);

    dlmwrite(['pop_stg_eq_',num2str(k),'.dat'],pop_stg_eq,'precision','%8.6f');

end  % Repeated with different initials

ret_info = 1;

end
```

soc_dis_sim.m:

```
function [ind_stg_eq,pop_stg_eq] = soc_dis_sim (ind_stg,pop_stg,A,w)

%
% Simulation of collective behavior of social distancing
%
% m individuals to visit n social sites:
%
% A -- connectivity matrix of social activities, n x n, n = 10
% w -- contact weights assigned to social activities, n x 1
%
% ind_stg -- strategies of individuals, n x m, m = 100
% pop_stg -- strategy of population, n x 1
%
% ind_stg_eq -- equilibrium strategies of individuals, n x m
% pop_stg_eq -- equilibrium strategy of population, n x 1
%
% r -- rationality, 0 - random, 1 - complete rational
% u -- # random behaviors allowed
% v -- # random behaviors prohibited
%
% Zhijun Wu, 12/20/2020, Math Dept, Iowa State University
%

W = diag(w);
A = (A*W + W*A) / 2;

[n,m] = size(ind_stg);

ind_stg_eq = ind_stg;
pop_stg_eq = pop_stg;

plot(ind_stg_eq,'ob','MarkerSize',8);
hold;

plot(pop_stg_eq,'*r','MarkerSize',8);

title('Generation 0','FontSize',16);
xlabel('Social Activities','FontSize',16,'FontWeight','Bold');
ylabel('Participating Frequencies','FontSize',16,'FontWeight','Bold');

hold;
pause;

% Initial and maximum # iterations

k = 0; K = 100;
```

```
d = zeros(K,1);

% Max payoff difference

con_max_0 = 1.0;

while (con_max_0 > 1.0e-12 && k < K)

  con_max_0 = 0;

  for j = 1 : m

     x = ind_stg_eq(1:n,j);
     y = pop_stg_eq;

     ind_stg_eq(1:n,j) = soc_dis_upd (x,y,A);

     pop_stg_eq = y + (ind_stg_eq(1:n,j) - x) / m;

     con_pop = A*pop_stg_eq;

     con_ind_eq(j) = ind_stg_eq(1:n,j)'*con_pop;
     con_pop_eq = pop_stg_eq'*con_pop;

     con_rel_eq = con_pop_eq * ones(n,1) - con_pop;

     con_ave = norm (con_rel_eq);

     if (con_ave > con_max_0)
        con_max_0 = con_ave;
     end

  end

  k = k + 1;

  if (mod(k,20) == 0)

  plot(ind_stg_eq,'ob','MarkerSize',8);
  hold;

  plot(pop_stg_eq,'*r','MarkerSize',8);

  title(['Generation ',num2str(k)],'FontSize',16);
  xlabel('Social Activities','FontSize',16,'FontWeight','Bold');
  ylabel('Participating Frequencies','FontSize',16,'FontWeight','Bold');
```

```
  hold;
  pause;

  end

  e = ones(m,1);
  c = sqrt(sum((ind_stg_eq - pop_stg_eq*e').^2));
  c = c';

  d(k,1) = sum(c) / m;

  p(k) = con_pop_eq;

  q(k,1:m) = con_ind_eq';

end

  pause;

  plot(d(1:k,1),'-b','LineWidth',2);
  hold;

  xdata = 1:1:k; xdata = xdata';
  ydata = d(1:k,1);

  x0 = [1;1;20;3];
  x1 = lsqcurvefit(@myfun,x0,xdata,ydata);
  ydata = myfun(x1,xdata);

  plot(xdata,ydata,'-r','LineWidth',4);

  title('Average Deviations of Individual Strategies','FontSize',16);
  xlabel('Generations','FontSize',16,'FontWeight','bold');
  ylabel('Average Deviations','FontSize',16,'FontWeight','bold');

  hold;
  pause;

end

function ydata = myfun(x,xdata)

ydata = -x(1)*atan(x(2)*(xdata - x(3))) + x(4);

end
```

soc_dis_upd.m:

```
function ind_stg_out = soc_dis_upd (ind_stg_in,pop_stg_in,A)

%
% Update of individual distancing strategy
%
% ind_stg_in -- current individual strategy, n x 1
% pop_stg_in -- current population strategy, n x 1
%
% A -- weighted connectivity matrix of social activities, n x n
%
% ind_stg_out -- updated individual strategy, n x 1
%
% Zhijun Wu, 12/20/2020, Math Dept, Iowa State University
%

n = size(ind_stg_in,1);

x = ind_stg_in;
y = pop_stg_in;

con_ind = A*y;
con_pop = y' * con_ind;

con_rel = con_pop * ones(n,1) - con_ind;

  for i = 1 : n

     %strategy i has lower contact, increase its frequency

     if (con_rel(i) > 0)
        if (x(i) < y(i))
           x(i) = x(i) + 1.0 * (y(i) - x(i));
        else
           x(i) = x(i) + 0.5 * min(x(i)-y(i),1.0-x(i));
        end
     end

     %strategy i has higher contact, reduce its frequency:

     if (con_rel(i) < 0)
        if (x(i) > y(i))
           x(i) = x(i) - 1.0 * (x(i) - y(i));
        else
           x(i) = x(i) - 0.5 * min(y(i)-x(i),x(i)-0.0);
        end
     end
```

```
  end
ind_stg_out = x / sum(x);
end
```
