# Supplementary material for: Social distancing is a social dilemma game played by every individual against his/her population
Source: PLoS One. 2021 Aug 2;16(8):e0255543. doi: 10.1371/journal.pone.0255543 (PMC8328347; doi:10.1371/journal.pone.0255543)
Supplement: S6 File — Code for simulation for a small multi-population with 10 social activities. (PDF) [file pone.0255543.s006.pdf]

S6 File. Simulation Code 2: Matlab code for simulation on small multipopulations.

README:

Simulation for the population in Fig 2 with two subpopulations:

A1.dat, w1.dat -- connectivity matrix and weights when activities are dependent.

A2.dat, w2.dat -- connectivity matrix and weights when activities are independent.

Copy them to A.dat and w.dat

Inside test_run_1.m, h1 and h2 need to be set with activity sets 1.

Simulation for a population that forms a Petersen's diagram:

A3.dat, w3.dat -- connectivity matrix and weights.

Copy them to A.dat and w.dat

Inside test_run_1.m, h1 and h2 need to be set with activity sets 2

In Matlab, type:

>test_run_1

There are a lot of pauses in the code so you can look the intermediate results and figures. Hit any key once or twice to continue.

Ending frequencies are saved in pop_stg_eq_1_*.dat for subpopulation 1 and in pop_stg_eq_2_*.dat for subpopulation 2.

A1.dat:

```
1 1 1 1 0 1 0 0 0 0
1 1 1 1 0 0 0 0 1 0
1 1 1 1 0 1 0 0 0 0
1 1 1 1 0 0 0 0 1 0
0 0 0 0 1 1 1 0 0 0
1 0 1 0 1 1 1 0 0 0
0 0 0 0 1 1 1 0 0 0
0 0 0 0 0 0 0 1 1 1
0 1 0 1 0 0 0 1 1 1
0 0 0 0 0 0 0 1 1 1
```

w1.dat:

4 4 4 4 1 1 1 1 1 1

A2.dat:

1 0 0 0 0 0 0 0 0 0
0 1 0 0 0 0 0 0 0 0
0 0 1 0 0 0 0 0 0 0
0 0 0 1 0 0 0 0 0 0
0 0 0 0 1 0 0 0 0 0
0 0 0 0 0 1 0 0 0 0
0 0 0 0 0 0 1 0 0 0
0 0 0 0 0 0 0 1 0 0
0 0 0 0 0 0 0 0 1 0
0 0 0 0 0 0 0 0 0 1

w2.dat:

4 4 4 4 1 1 1 1 1 1

A3.dat:

1 1 0 0 1 1 0 0 0 0
1 1 1 0 0 0 1 0 0 0
0 1 1 1 0 0 0 1 0 0
0 0 1 1 1 0 0 0 1 0
1 0 0 1 1 0 0 0 0 1
1 0 0 0 0 1 0 1 1 0
0 1 0 0 0 0 1 0 1 1
0 0 1 0 0 1 0 1 0 1
0 0 0 1 0 1 1 0 1 0
0 0 0 0 1 0 1 1 0 1

w3.dat:

4 4 4 4 4 1 1 1 1 1

test_run_1.m:

```
function [ret_info,ind_stg_eq_1,pop_stg_eq_1,ind_stg_eq_2,pop_stg_eq_2] = test_run_1 ()

%
% Simulation of collective behavior of social distancing
%
% (Small, multi-populations)
%
% m -- # total individuals in population, m = 100
%
% m1 -- #individuals in subpop 1
% m2 -- #individuals in subpop 2
%
% A -- connectivity matrix of social activities, n x n, n = 85
% w -- contact weights assigned to social activities, n x 1
%
% ind_stg_in_1 -- initial strategies of individuals in subpop 1, n x m1
% pop_stg_in_1 -- initial strategy of subpop 1, n x 1
%
% ind_stg_in_2 -- initial strategies of individuals in subpop 2, n x m2
% pop_stg_in_2 -- initial strategy of subpop 2, n x 1
%
% ind_stg_eq_1 -- equilibrium strategies of individuals in subpop 1, n x m1
% pop_stg_eq_1 -- equilibrium strategy of subpop 1, n x 1
%
% ind_stg_eq_2 -- equilibrium strategies of individuals in subpop 2, n x m2
% pop_stg_eq_2 -- equilibrium strategy of subpop 2, n x 1
%
% ret_info -- 1 -- suceeds, 0 -- fails
%
% Zhijun Wu, 12/20/2020, Math Dept, Iowa State University
%

% Load contact matrix and contact weights

A = load('A.dat','-ascii');
w = load('w.dat','-ascii');

k = size(A,1);

% Obtain # strategies and # individuals

n = k;
m = 10*n;

m1 = m/2;
m2 = m - m1;
```

```
% activity sets for subpop 1 and sub pop 2

%
% Subpopulation activity sets 1
%
 h1 = [1,1,1,1,1,1,1,0,0,0]';
 h2 = [1,1,1,1,0,0,0,1,1,1]';

% Subpopulation activity sets 2
%
% h1 = [1,1,1,1,1,1,1,0,1,0]';
% h2 = [1,1,1,1,1,0,0,1,0,1]';


ind_stg_in_1 = zeros(n,m1);
pop_stg_in_1 = zeros(n,1);

ind_stg_in_2 = zeros(n,m2);
pop_stg_in_2 = zeros(n,1);

K = 100;

% Start with initial random strategies

rng ('default');

for i = 0 : n-1          % Repeat with different initials

   for j = 0 : n-1
      k = rem(i+j,n)+1;
      ind_stg_in_1(k,1:m1) = (j+1)*rand(1,m1);
      ind_stg_in_2(k,1:m2) = (j+1)*rand(1,m2);
   end

   for l = 1 : m1
      ind_stg_in_1(1:n,l) = h1.*ind_stg_in_1(1:n,l);
   end

   for l = 1 : m1
      ind_stg_in_1(1:n,l) = ind_stg_in_1(1:n,l) / sum(ind_stg_in_1(1:n,l));
   end

   for l = 1 : m2
      ind_stg_in_2(1:n,l) = h2.*ind_stg_in_2(1:n,l);
   end

   for l = 1 : m2
```

```
        ind_stg_in_2(1:n,l) = ind_stg_in_2(1:n,l) / sum(ind_stg_in_2(1:n,l));
    end

    for l = 1 : n
        pop_stg_in_1(l) = sum(ind_stg_in_1(l,1:m1)) / m1;
    end

    for l = 1 : n
        pop_stg_in_2(l) = sum(ind_stg_in_2(l,1:m2)) / m2;
    end

    % Start simulation, to reach equilibrium strategies

    [ind_stg_eq_1,pop_stg_eq_1,ind_stg_eq_2,pop_stg_eq_2] = soc_dis_sim
(ind_stg_in_1,pop_stg_in_1,h1,ind_stg_in_2,pop_stg_in_2,h2,A,w);

    dlmwrite(['pop_stg_eq_1_',num2str(k),'.dat'],pop_stg_eq_1,'precision','%8.6f');
    dlmwrite(['pop_stg_eq_2_',num2str(k),'.dat'],pop_stg_eq_2,'precision','%8.6f');

end  % Repeated with different initials

ret_info = 1;

end
```

soc_dis_sim.m:

```
function [ind_stg_eq_1,pop_stg_eq_1,ind_stg_eq_2,pop_stg_eq_2] = soc_dis_sim
(ind_stg_in_1,pop_stg_in_1,act_set_1,ind_stg_in_2,pop_stg_in_2,act_set_2,A,w)

%
% Simulation of collective behavior of social distancing
%
% A -- connectivity matrix of social activities, n x n, n = 10
% w -- contact weights assigned to social activities, n x 1
%
% ind_stg_in_1 -- initial strategies of individuals in subpop 1, n x m1
% pop_stg_in_1 -- initial strategy of subpop 1, n x 1
%
% ind_stg_in_2 -- initial strategies of individuals in subpop 2, n x m2
% pop_stg_in_2 -- initial strategy of subpop 2, n x 1
%
% ind_stg_eq_1 -- equilibrium strategies of individuals in subpop 1, n x m1
% pop_stg_eq_1 -- equilibrium strategy of subpop 1, n x 1
%
% ind_stg_eq_2 -- equilibrium strategies of individuals in subpop 2, n x m2
% pop_stg_eq_2 -- equilibrium strategy of subpop 2, n x 1
%
% act_set_1 -- activity set for subpop 1
% act_set_2 -- activity set for subpop 2
%
% Zhijun Wu, 12/20/2020, Math Dept, Iowa State University
%

W = diag(w);
A = (A*W + W*A) / 2;

n = size(ind_stg_in_1,1);

m1 = size(ind_stg_in_1,2);
m2 = size(ind_stg_in_2,2);

ind_stg_eq_1 = ind_stg_in_1;
pop_stg_eq_1 = pop_stg_in_1;

ind_stg_eq_2 = ind_stg_in_2;
pop_stg_eq_2 = pop_stg_in_2;

H1 = figure;

plot(ind_stg_eq_1,'ob','MarkerSize',8);
hold;
```

```
plot(pop_stg_eq_1,'*r','MarkerSize',8);

title('Generation 0','FontSize',16);
xlabel('Social Activities','FontSize',16,'FontWeight','Bold');
ylabel('Participating Frequencies','FontSize',16,'FontWeight','Bold');

hold;

H2 = figure;

plot(ind_stg_eq_2,'ob','MarkerSize',8);
hold;

plot(pop_stg_eq_2,'*r','MarkerSize',8);

title('Generation 0','FontSize',16);
xlabel('Social Activities','FontSize',16,'FontWeight','Bold');
ylabel('Participating Frequencies','FontSize',16,'FontWeight','Bold');

hold;

pause;

% Initial and maximum # iterations

k = 0; K = 100;

d = zeros(K,1);

% Max payoff difference

max_dev = 1;

x1 = zeros(n,1);
y1 = pop_stg_eq_1;

x2 = zeros(n,1);
y2 = pop_stg_eq_2;

h1 = act_set_1;
h2 = act_set_2;

while (max_dev > 1.0e-8 && k < K)

  for j = 1 : m1

     x1 = ind_stg_eq_1(1:n,j);
     y1 = pop_stg_eq_1;
```

```
    ind_stg_eq_1(1:n,j) = soc_dis_upd (x1,y1,h1,y2,h2,A);

    for l = 1 : n
        pop_stg_eq_1(l) = y1(l) + (ind_stg_eq_1(l,j) - x1(l)) / m1;
    end

end

for j = 1 : m2

    x2 = ind_stg_eq_2(1:n,j);
    y2 = pop_stg_eq_2;

    ind_stg_eq_2(1:n,j) = soc_dis_upd (x2,y2,h2,y1,h1,A);

    for l = 1 : n
        pop_stg_eq_2(l) = y2(l) + (ind_stg_eq_2(l,j) - x2(l)) / m2;
    end

end

k = k + 1;

e = ones(n,1);

e1 = ones(m1,1);
c1 = sqrt(sum(((ind_stg_eq_1 - pop_stg_eq_1*e1').*(h1*e1')).^2));
d1 = sum(c1) / m1;

e2 = ones(m2,1);
c2 = sqrt(sum(((ind_stg_eq_2 - pop_stg_eq_2*e2').*(h2*e2')).^2));
d2 = sum(c2) / m2;

d(k) = (d1 + d2) / 2;

max_dev = max(d);

if (mod(k,20) == 0)

figure(H1);

plot(ind_stg_eq_1,'ob','MarkerSize',8);
hold;

plot(pop_stg_eq_1,'*r','MarkerSize',8);

title(['Generation ',num2str(k)],'FontSize',16);
```

```
  xlabel('Social Activities','FontSize',16,'FontWeight','Bold');
  ylabel('Participating Frequencies','FontSize',16,'FontWeight','Bold');

  hold;

  figure(H2);

  plot(ind_stg_eq_2,'ob','MarkerSize',8);
  hold;

  plot(pop_stg_eq_2,'*r','MarkerSize',8);

  title(['Generation ',num2str(k)],'FontSize',16);
  xlabel('Social Activities','FontSize',16,'FontWeight','Bold');
  ylabel('Participating Frequencies','FontSize',16,'FontWeight','Bold');

  hold;
  pause;

  end

end

  pause;

  H3 = figure;

  plot(d(1:k,1),'-b','LineWidth',2);
  hold;

  xdata = 1:1:k; xdata = xdata';
  ydata = d(1:k,1);

    x0 = [1;1;20;3];
    x1 = lsqcurvefit(@myfun,x0,xdata,ydata);
    ydata = myfun(x1,xdata);

  plot(xdata,ydata,'-r','LineWidth',4);

  title('Average Deviations of Individual Strategies','FontSize',16);
  xlabel('Generations','FontSize',16,'FontWeight','bold');
  ylabel('Average Deviations','FontSize',16,'FontWeight','bold');

  hold;
  pause;

  close all;
```

```
end

function ydata = myfun(x,xdata)

ydata = -x(1)*atan(x(2)*(xdata - x(3))) + x(4);

end
```

soc_dis_upd.m:

```
function ind_stg_out_1 = soc_dis_upd (ind_stg_in_1,pop_stg_in_1,act_set_1,pop_stg_in_2,act_set_2,A)

%
% Update of individual distancing strategy
%
% A -- weighted connectivity matrix of social activities, n x n, n = 10
%
% ind_stg_in_1 -- initial strategies of individuals in subpop 1, n x m1
% pop_stg_in_1 -- initial strategy of subpop 1, n x 1
%
% pop_stg_in_2 -- initial strategy of subpop 2, n x 1
%
% ind_stg_out_1 -- equilibrium strategies of individuals in subpop 1, n x m1
%
% act_set_1 -- activity set for subpop 1
% act_set_2 -- activity set for subpop 2
%
% Zhijun Wu, 12/20/2020, Math Dept, Iowa State University
%

n = size(ind_stg_in_1,1);

x1 = ind_stg_in_1;

y1 = pop_stg_in_1;
h1 = act_set_1;

y2 = pop_stg_in_2;
h2 = act_set_2;

max_con = (A*(y1 + y2)).*h1;
ave_con = y1' * max_con;

rel_con = ave_con .* h1 - max_con;

  for i = 1 : n

     if (h1(i) == 1)

     %strategy i has lower contact, increase its frequency

     if (rel_con(i) > 0)
        if (x1(i) < y1(i))
           x1(i) = x1(i) + 1.0 * (y1(i) - x1(i));
        else
           x1(i) = x1(i) + 0.5 * min(x1(i)-y1(i),1.0-x1(i));
```

```
      end
    end

    %strategy i has higher contact, reduce its frequency:

    if (rel_con(i) < 0)
      if (x1(i) > y1(i))
        x1(i) = x1(i) - 1.0 * (x1(i) - y1(i));
      else
        x1(i) = x1(i) - 0.5 * min(y1(i)-x1(i),x1(i)-0.0);
      end
    end

    end

  end

ind_stg_out_1 = x1 / sum(x1);

end
```
