# Supplementary material for: Social distancing is a social dilemma game played by every individual against his/her population
Source: PLoS One. 2021 Aug 2;16(8):e0255543. doi: 10.1371/journal.pone.0255543 (PMC8328347; doi:10.1371/journal.pone.0255543)
Supplement: S7 File — Code for simulation for a single large population in the small university town. (PDF) [file pone.0255543.s007.pdf]

S7 File. Simulation Code 3: Matlab code for simulation on large single populations.

README:

Simulation for the population in Fig 5:

A.dat, w.dat -- generated by A_generate.m and w_generate.m.

In Matlab, type:

>test_run_1

There are a lot of pauses in the code so you can look the intermediate results and figures. Hit any key once or twice to continue.

Ending frequencies are saved in pop_stg_eq_*.dat.

A_generate.m:

```
function A = A_generate ()

%
% Simulation of collective behavior of social distancing
%
% A -- connectivity matrix of social activities, n x n, n = 85
%
%
% Zhijun Wu, 12/20/2020, Math Dept, Iowa State University
%

n = 85;

A = eye(n);

% College Campus

A(1,2) = 1; A(1,3) = 1; A(1,4) = 1; A(1,5) = 1;
A(2,1) = 1; A(2,3) = 1; A(2,4) = 1; A(2,6) = 1;
A(3,1) = 1; A(3,2) = 1; A(2,4) = 1; A(3,7) = 1; A(3,9) = 1;
A(4,1) = 1; A(4,2) = 1; A(4,3) = 1; A(4,8) = 1; A(4,10) = 1;

A(5,1) = 1;
A(6,2) = 1;
A(7,3) = 1;
A(8,4) = 1;
```

```
% College Town

A(9,3) = 1; A(9,10) = 1; A(9,11) = 1;
A(10,4) = 1; A(10,9) = 1; A(10,12) = 1;
A(11,9) = 1; A(11,12) = 1; A(11,13) = 1;
A(12,10) = 1; A(12,11) = 1; A(12,14) = 1;
A(13,11) = 1; A(13,14) = 1;
A(14,12) = 1; A(14,13) = 1;

% W HyVee

A(15,16) = 1; A(15,17) = 1; A(15,19) = 1;
A(16,15) = 1; A(16,18) = 1; A(16,20) = 1;
A(17,15) = 1; A(17,18) = 1; A(17,21) = 1; A(17,23) = 1;
A(18,16) = 1; A(18,17) = 1; A(18,22) = 1; A(18,24) = 1;

A(19,15) = 1;
A(20,16) = 1;
A(21,17) = 1;
A(22,18) = 1;
A(23,17) = 1;
A(24,18) = 1;

% Somerset

A(25,26) = 1; A(25,27) = 1; A(25,28) = 1;
A(26,25) = 1; A(26,27) = 1; A(26,28) = 1;
A(27,25) = 1; A(27,26) = 1; A(27,28) = 1;
A(28,25) = 1; A(28,26) = 1; A(28,27) = 1;

% N Residential

A(29,30) = 1; A(29,31) = 1;
A(30,29) = 1;
A(31,29) = 1; A(31,32) = 1;
A(32,31) = 1;

% N Dakota

A(33,34) = 1; A(33,35) = 1;
A(34,33) = 1; A(34,35) = 1;
A(35,33) = 1; A(35,34) = 1;

% S Dakota

A(36,37) = 1; A(36,38) = 1;
A(37,36) = 1; A(37,38) = 1;
A(38,36) = 1; A(38,37) = 1;
```

% S Residential

A(39,40) = 1; A(39,41) = 1;
A(40,39) = 1; A(40,41) = 1;
A(41,39) = 1; A(41,40) = 1;

% E Residential

A(42,43) = 1; A(42,44) = 1;
A(43,42) = 1; A(43,44) = 1;
A(44,42) = 1; A(44,43) = 1;

% N Grand Mall

A(45,47) = 1; A(45,51) = 1; A(45,53) = 1; A(45,58) = 1;
A(46,48) = 1; A(46,52) = 1; A(46,54) = 1; A(46,63) = 1;
A(47,45) = 1; A(47,49) = 1; A(47,51) = 1; A(47,53) = 1;
A(48,46) = 1; A(48,50) = 1; A(48,52) = 1; A(48,54) = 1;

A(49,47) = 1; A(49,55) = 1;
A(50,48) = 1; A(50,56) = 1;

A(51,45) = 1; A(51,47) = 1; A(51,52) = 1; A(51,53) = 1; A(51,54) = 1;
A(52,46) = 1; A(52,48) = 1; A(52,51) = 1; A(52,53) = 1; A(52,54) = 1;
A(53,45) = 1; A(53,47) = 1; A(53,51) = 1; A(53,52) = 1; A(53,54) = 1;
A(54,46) = 1; A(54,48) = 1; A(54,51) = 1; A(54,52) = 1; A(54,53) = 1;

A(55,49) = 1; A(55,56) = 1;
A(56,50) = 1; A(56,55) = 1;

% N Lights

A(57,58) = 1;
A(58,45) = 1; A(58,57) = 1; A(58,59) = 1;
A(59,58) = 1; A(59,60) = 1;
A(60,59) = 1; A(60,61) = 1;
A(61,60) = 1; A(61,62) = 1;
A(62,61) = 1; A(62,63) = 1; A(62,64) = 1;
A(63,46) = 1; A(63,62) = 1; A(63,65) = 1;

A(64,62) = 1; A(64,65) = 1; A(64,66) = 1; A(64,67) = 1;
A(65,63) = 1; A(65,64) = 1; A(65,66) = 1; A(65,67) = 1;
A(66,64) = 1; A(66,65) = 1; A(66,67) = 1;
A(67,64) = 1; A(67,65) = 1; A(67,66) = 1;

% Commercial

```
A(68,69) = 1; A(68,80) = 1;
A(69,68) = 1; A(69,70) = 1;
A(70,69) = 1; A(70,71) = 1;
A(71,70) = 1; A(71,72) = 1;

A(72,71) = 1; A(72,73) = 1; A(72,76) = 1;
A(73,72) = 1; A(73,74) = 1; A(73,77) = 1;

A(74,73) = 1; A(74,75) = 1;
A(75,74) = 1; A(75,81) = 1;

% E Commercial

A(76,72) = 1; A(76,77) = 1; A(76,78) = 1;
A(77,73) = 1; A(77,76) = 1; A(77,79) = 1;
A(78,76) = 1; A(78,79) = 1;
A(79,77) = 1; A(79,78) = 1;

A(80,68) = 1;
A(81,75) = 1;

% S Commercial

A(82,83) = 1; A(82,84) = 1;
A(83,82) = 1; A(83,85) = 1;
A(84,82) = 1; A(84,85) = 1;
A(85,83) = 1; A(85,84) = 1;

writematrix(A,'A.dat','Delimiter',' ');

end
```

w_generate.m:

```
function w = w_generate ()

%
% Simulation of collective behavior of social distancing
%
% w -- contact weights assigned to social activities, n x 1
%
%
% Zhijun Wu, 12/20/2020, Math Dept, Iowa State University
%

n = 85;

w = zeros(n,1);

% College Campus

w(1) = 6; w(2) = 6; w(3) = 6; w(4) = 6;
w(5) = 6; w(6) = 6; w(7) = 6; w(8) =6;

% College Town

w(9) = 6; w(10) = 6; w(11) = 6; w(12) = 6; w(13) = 6; w(14) = 6;

% W HyVee

w(15) = 2; w(16) = 2; w(17) = 2; w(18) = 2;
w(19) = 2; w(20) = 2; w(21) = 2; w(22) = 2; w(23) = 2; w(24) = 2;

% Somerset

w(25) = 4; w(26) = 4; w(27) = 4; w(28) = 4;

% Residential Areas

w(29) = 1; w(30) = 1; w(31) = 1; w(32) = 1;

w(33) = 1; w(34) = 1; w(35) = 1;
w(36) = 1; w(37) = 1; w(38) = 1;
w(39) = 1; w(40) = 1; w(41) = 1;
w(42) = 1; w(43) = 1; w(44) = 1;

% N Grand Mall

w(45) = 4; w(46) = 4; w(47) = 4; w(48) = 4;
w(49) = 2; w(50) = 2;
```

```
w(51) = 4; w(52) = 4; w(53) = 4; w(54) = 4;
w(55) = 2; w(56) = 2;

% N Lights

w(57) = 2; w(58) = 2; w(59) = 2;
w(60) = 2; w(61) = 2; w(62) = 2; w(63) = 2;
w(64) = 4; w(65) = 4; w(66) = 4; w(67) = 4;

% Commercial Areas

w(68) = 4; w(69) = 4; w(70) = 4; w(71) = 4;
w(72) = 4; w(73) = 4; w(74) = 4; w(75) = 4;

w(76) = 2; w(77) = 2; w(78) = 2; w(79) = 2;
w(80) = 4; w(81) = 4;

w(82) = 4; w(83) = 4; w(84) = 4; w(85) = 4;

writematrix(w,'w.dat','Delimiter',' ');

end
```

test_run_1.m:

```
function [ret_info,ind_stg_eq,pop_stg_eq] = test_run_1 ()

%
% Simulation of collective behavior of social distancing
%
% (Large, single population)
%
% m # individuals in population, m = 850
%
% A -- connectivity matrix of social activities, n x n, n = 85
% w -- contact weights assigned to social activities, n x 1
%
% ind_stg_in -- initial strategies of individuals, n x m
% pop_stg_in -- initial strategy of population, n x 1
%
% ind_stg_eq -- equilibrium strategies of individuals, n x m
% pop_stg_eq -- equilibrium strategy of population, n x 1
%
% ret_info -- 1 -- suceeds, 0 -- fails
%
% Zhijun Wu, 12/20/2020, Math Dept, Iowa State University
%

% Load contact matrix and contact weights

A = load('A.dat','-ascii');
w = load('w.dat','-ascii');

k = size(A,1);

% Obtain # strategies and # individuals

n = k;
m = 10*k;

% Start with initial random strategies

rng ('default');

for k = 1 : 10          % Repeat with different initials

   ind_stg_in = rand(n,m);

   for l = 1 : m
      ind_stg_in(1:n,l) = ind_stg_in(1:n,l) / sum(ind_stg_in(1:n,l));
   end
```

```
    pop_stg_in = sum(ind_stg_in,2) / m;

    % Start simulation, to reach equilibrium strategies

    [ind_stg_eq,pop_stg_eq] = soc_dis_sim (ind_stg_in,pop_stg_in,A,w);

    dlmwrite(['pop_stg_eq_',num2str(k),'.dat'],pop_stg_eq,'precision','%8.6f');

end  % Repeated with different initials

ret_info = 1;

end
```

soc_dis_sim.m:

```
function [ind_stg_eq,pop_stg_eq] = soc_dis_sim (ind_stg,pop_stg,A,w)

%
% Simulation of collective behavior of social distancing
%
% m individuals to participate n social activities:
%
% A -- connectivity matrix of social activities, n x n, n = 85
% w -- contact weights assigned to social activities, n x 1
%
% ind_stg -- strategies of individuals, n x m
% pop_stg -- strategy of population, n x 1
%
% ind_stg_eq -- equilibrium strategies of individuals, n x m
% pop_stg_eq -- equilibrium strategy of population, n x 1
%
% Zhijun Wu, 12/20/2020, Math Dept, Iowa State University
%

W = diag(w);
A = (A*W + W*A) / 2;

[n,m] = size(ind_stg);

ind_stg_eq = ind_stg;
pop_stg_eq = pop_stg;

plot(ind_stg_eq,'ob','MarkerSize',4);
hold;

plot(pop_stg_eq,'*r','MarkerSize',4);

title('Generation 0','FontSize',16);
xlabel('Social Activities','FontSize',16,'FontWeight','Bold');
ylabel('Participating Frequencies','FontSize',16,'FontWeight','Bold');

hold;
pause;

% Initial and maximum # iterations

k = 0; K = 400;

% Averaged RMSD

d = zeros(K,1);
```

```
% Max payoff difference

con_max_0 = 1;

while (con_max_0 > 1.0e-12 && k < K)

  con_max_0 = 0;

  for j = 1 : m

     x = ind_stg_eq(1:n,j);
     y = pop_stg_eq;

     ind_stg_eq(1:n,j) = soc_dis_upd (x,y,A);

     pop_stg_eq = y + (ind_stg_eq(1:n,j) - x) / m;

     con_pop = A*pop_stg_eq;

     con_ind_eq(j) = ind_stg_eq(1:n,j)'*con_pop;
     con_pop_eq = pop_stg_eq'*con_pop;

     con_rel_eq = con_pop_eq * ones(n,1) - con_pop;

     con_ave = norm (con_rel_eq);

     if (con_ave > con_max_0)
       con_max_0 = con_ave;
     end

  end

  k = k + 1;

  if (mod(k,40) == 0)

  plot(ind_stg_eq,'ob','MarkerSize',4);
  hold;

  plot(pop_stg_eq,'*r','MarkerSize',4);

  title(['Generation ',num2str(k)],'FontSize',16);
  xlabel('Social Activities','FontSize',16,'FontWeight','Bold');
  ylabel('Participating Frequencies','FontSize',16,'FontWeight','Bold');

  hold;
  pause;
```

```
  end

  e = ones(m,1);
  c = sqrt(sum((ind_stg_eq - pop_stg_eq*e').^2));
  c = c';

  d(k,1) = sum(c) / m;

end

  pause;

  plot(d(1:k,1),'-b','LineWidth',2);
  hold;

  xdata = 1:1:k; xdata = xdata';
  ydata = d(1:k,1);

  x0 = [1;1;50;3];
  x1 = lsqcurvefit(@myfun,x0,xdata,ydata);
  ydata = myfun(x1,xdata);

  plot(xdata,ydata,'-r','LineWidth',4);

  title('Average Deviations of Individual Strategies','FontSize',16);
  xlabel('Generations','FontSize',16,'FontWeight','bold');
  ylabel('Average Deviations','FontSize',16,'FontWeight','bold');

  hold;
  pause;

end

function ydata = myfun(x,xdata)

ydata = -x(1)*atan(x(2)*(xdata - x(3))) + x(4);

end
```

soc_dis_upd.m:

```
function ind_stg_out = soc_dis_upd (ind_stg_in,pop_stg_in,A)

%
% Update of individual distancing strategy
%
% ind_stg_in -- current individual strategy, n x 1
% pop_stg_in -- current population strategy, n x 1
%
% A -- weighted connectivity matrix of social activities, n x n, n = 85
%
% ind_stg_out -- updated individual strategy, n x 1
%
% Zhijun Wu, 12/20/2020, Math Dept, Iowa State University
%

n = size(ind_stg_in,1);

x = ind_stg_in;
y = pop_stg_in;

con_max = A*y;
con_ave = y' * con_ind;

con_rel = con_ave * ones(n,1) - con_max;

for i = 1 : n

   %strategy i has lower contact, increase its frequency

   if (con_rel(i) > 0)
      if (x(i) < y(i))
         x(i) = x(i) + 1.0 * (y(i) - x(i));
      else
         x(i) = x(i) + 0.5 * min(x(i)-y(i),1.0-x(i));
      end
   end

   %strategy i has higher contact, reduce its frequency:

   if (con_rel(i) < 0)
      if (x(i) > y(i))
         x(i) = x(i) - 1.0 * (x(i) - y(i));
      else
         x(i) = x(i) - 0.5 * min(y(i)-x(i),x(i)-0.0);
      end
   end
```

```
end

ind_stg_out = x / sum(x);

end
```
