# Supplementary material for: Social distancing is a social dilemma game played by every individual against his/her population
Source: PLoS One. 2021 Aug 2;16(8):e0255543. doi: 10.1371/journal.pone.0255543 (PMC8328347; doi:10.1371/journal.pone.0255543)
Supplement: S8 File — Code for simulation for a large multi-population in the small university town. (PDF) [file pone.0255543.s008.pdf]

S8 File. Simulation Code 4: Matlab code for simulation on large multipopulations.

README:

Simulation for the population in Fig 5 with six subpopulations:

A.dat, w.dat -- connectivity matrix and weights generated by A_generate.m and w_generate.m

h.dat -- activity sets for the subpopulations, generated by h_generate.m

In Matlab, type:

>test_run_1

There are a lot of pauses in the code so you can look the intermediate results and figures. Hit any key once or twice to continue.

Ending frequencies are saved in pop_stg_eq_*.dat for all subpopulations.

A_generate.m:

```
function A = A_generate ()

%
% Simulation of collective behavior of social distancing
%
% A -- connectivity matrix of social activities, n x n, n = 85
%
%
% Zhijun Wu, 12/20/2020, Math Dept, Iowa State University
%

n = 85;

A = eye(n);

% College Campus

A(1,2) = 1; A(1,3) = 1; A(1,4) = 1; A(1,5) = 1;
A(2,1) = 1; A(2,3) = 1; A(2,4) = 1; A(2,6) = 1;
A(3,1) = 1; A(3,2) = 1; A(2,4) = 1; A(3,7) = 1; A(3,9) = 1;
A(4,1) = 1; A(4,2) = 1; A(4,3) = 1; A(4,8) = 1; A(4,10) = 1;

A(5,1) = 1;
A(6,2) = 1;
A(7,3) = 1;
```

```
A(8,4) = 1;

% College Town

A(9,3) = 1; A(9,10) = 1; A(9,11) = 1;
A(10,4) = 1; A(10,9) = 1; A(10,12) = 1;
A(11,9) = 1; A(11,12) = 1; A(11,13) = 1;
A(12,10) = 1; A(12,11) = 1; A(12,14) = 1;
A(13,11) = 1; A(13,14) = 1;
A(14,12) = 1; A(14,13) = 1;

% W HyVee

A(15,16) = 1; A(15,17) = 1; A(15,19) = 1;
A(16,15) = 1; A(16,18) = 1; A(16,20) = 1;
A(17,15) = 1; A(17,18) = 1; A(17,21) = 1; A(17,23) = 1;
A(18,16) = 1; A(18,17) = 1; A(18,22) = 1; A(18,24) = 1;

A(19,15) = 1;
A(20,16) = 1;
A(21,17) = 1;
A(22,18) = 1;
A(23,17) = 1;
A(24,18) = 1;

% Somerset

A(25,26) = 1; A(25,27) = 1; A(25,28) = 1;
A(26,25) = 1; A(26,27) = 1; A(26,28) = 1;
A(27,25) = 1; A(27,26) = 1; A(27,28) = 1;
A(28,25) = 1; A(28,26) = 1; A(28,27) = 1;

% N Residential

A(29,30) = 1; A(29,31) = 1;
A(30,29) = 1;
A(31,29) = 1; A(31,32) = 1;
A(32,31) = 1;

% N Dakota

A(33,34) = 1; A(33,35) = 1;
A(34,33) = 1; A(34,35) = 1;
A(35,33) = 1; A(35,34) = 1;

% S Dakota

A(36,37) = 1; A(36,38) = 1;
```

A(37,36) = 1; A(37,38) = 1;
A(38,36) = 1; A(38,37) = 1;

% S Residential

A(39,40) = 1; A(39,41) = 1;
A(40,39) = 1; A(40,41) = 1;
A(41,39) = 1; A(41,40) = 1;

% E Residential

A(42,43) = 1; A(42,44) = 1;
A(43,42) = 1; A(43,44) = 1;
A(44,42) = 1; A(44,43) = 1;

% N Grand Mall

A(45,47) = 1; A(45,51) = 1; A(45,53) = 1; A(45,58) = 1;
A(46,48) = 1; A(46,52) = 1; A(46,54) = 1; A(46,63) = 1;
A(47,45) = 1; A(47,49) = 1; A(47,51) = 1; A(47,53) = 1;
A(48,46) = 1; A(48,50) = 1; A(48,52) = 1; A(48,54) = 1;

A(49,47) = 1; A(49,55) = 1;
A(50,48) = 1; A(50,56) = 1;

A(51,45) = 1; A(51,47) = 1; A(51,52) = 1; A(51,53) = 1; A(51,54) = 1;
A(52,46) = 1; A(52,48) = 1; A(52,51) = 1; A(52,53) = 1; A(52,54) = 1;
A(53,45) = 1; A(53,47) = 1; A(53,51) = 1; A(53,52) = 1; A(53,54) = 1;
A(54,46) = 1; A(54,48) = 1; A(54,51) = 1; A(54,52) = 1; A(54,53) = 1;

A(55,49) = 1; A(55,56) = 1;
A(56,50) = 1; A(56,55) = 1;

% N Lights

A(57,58) = 1;
A(58,45) = 1; A(58,57) = 1; A(58,59) = 1;
A(59,58) = 1; A(59,60) = 1;
A(60,59) = 1; A(60,61) = 1;
A(61,60) = 1; A(61,62) = 1;
A(62,61) = 1; A(62,63) = 1; A(62,64) = 1;
A(63,46) = 1; A(63,62) = 1; A(63,65) = 1;

A(64,62) = 1; A(64,65) = 1; A(64,66) = 1; A(64,67) = 1;
A(65,63) = 1; A(65,64) = 1; A(65,66) = 1; A(65,67) = 1;
A(66,64) = 1; A(66,65) = 1; A(66,67) = 1;
A(67,64) = 1; A(67,65) = 1; A(67,66) = 1;

```
% Commercial

A(68,69) = 1; A(68,80) = 1;
A(69,68) = 1; A(69,70) = 1;
A(70,69) = 1; A(70,71) = 1;
A(71,70) = 1; A(71,72) = 1;

A(72,71) = 1; A(72,73) = 1; A(72,76) = 1;
A(73,72) = 1; A(73,74) = 1; A(73,77) = 1;

A(74,73) = 1; A(74,75) = 1;
A(75,74) = 1; A(75,81) = 1;

% E Commercial

A(76,72) = 1; A(76,77) = 1; A(76,78) = 1;
A(77,73) = 1; A(77,76) = 1; A(77,79) = 1;
A(78,76) = 1; A(78,79) = 1;
A(79,77) = 1; A(79,78) = 1;

A(80,68) = 1;
A(81,75) = 1;

% S Commercial

A(82,83) = 1; A(82,84) = 1;
A(83,82) = 1; A(83,85) = 1;
A(84,82) = 1; A(84,85) = 1;
A(85,83) = 1; A(85,84) = 1;

writematrix(A,'A.dat','Delimiter',' ');

end
```

w_generate.m:

```
function w = w_generate ()

%
% Simulation of collective behavior of social distancing
%
% w -- contact weights assigned to social activities, n x 1
%
%
% Zhijun Wu, 12/20/2020, Math Dept, Iowa State University
%
```

```
n = 85;

w = zeros(n,1);

% College Campus

w(1) = 6; w(2) = 6; w(3) = 6; w(4) = 6;
w(5) = 6; w(6) = 6; w(7) = 6; w(8) =6;

% College Town

w(9) = 6; w(10) = 6; w(11) = 6; w(12) = 6; w(13) = 6; w(14) = 6;

% W HyVee

w(15) = 2; w(16) = 2; w(17) = 2; w(18) = 2;
w(19) = 2; w(20) = 2; w(21) = 2; w(22) = 2; w(23) = 2; w(24) = 2;

% Somerset

w(25) = 4; w(26) = 4; w(27) = 4; w(28) = 4;

% Residential Areas

w(29) = 1; w(30) = 1; w(31) = 1; w(32) = 1;

w(33) = 1; w(34) = 1; w(35) = 1;
w(36) = 1; w(37) = 1; w(38) = 1;
w(39) = 1; w(40) = 1; w(41) = 1;
w(42) = 1; w(43) = 1; w(44) = 1;

% N Grand Mall

w(45) = 4; w(46) = 4; w(47) = 4; w(48) = 4;
w(49) = 2; w(50) = 2;
w(51) = 4; w(52) = 4; w(53) = 4; w(54) = 4;
w(55) = 2; w(56) = 2;

% N Lights

w(57) = 2; w(58) = 2; w(59) = 2;
w(60) = 2; w(61) = 2; w(62) = 2; w(63) = 2;
w(64) = 4; w(65) = 4; w(66) = 4; w(67) = 4;

% Commercial Areas

w(68) = 4; w(69) = 4; w(70) = 4; w(71) = 4;
w(72) = 4; w(73) = 4; w(74) = 4; w(75) = 4;
```

```
w(76) = 2; w(77) = 2; w(78) = 2; w(79) = 2;
w(80) = 4; w(81) = 4;

w(82) = 4; w(83) = 4; w(84) = 4; w(85) = 4;

writematrix(w,'w.dat','Delimiter',' ');

end
```

h_generate.m:

```
function h = h_generate ()

%
% Simulation of collective behavior of social distancing
%
% h -- activity sets for subpopulations, n x M
%
% Zhijun Wu, 12/20/2020, Math Dept, Iowa State University
%

n = 85; M = 6;

h = zeros(n,M);

h(1:8,1) = 1; h(9:14,1) = 1; h(15:24) = 1; h(45:56,1) = 1;

h(1:8,2) = 1; h(25:28,2) = 1; h(29:32,2) = 1;
h(45:56,2) = 1; h(57:67,2) = 1; h(68:85,2) = 1;

h(1:8,3) = 1; h(15:24,3) = 1; h(25:28,3) = 1;
h(33:35,3) = 1; h(45:56,3) = 1; h(68:85,3) = 1;

h(1:8,4) = 1; h(15:24,4) = 1; h(25:28,4) = 1;
h(36:38,4) = 1; h(45:56,4) = 1; h(68:85,4) = 1;

h(1:8,5) = 1; h(15:24,5) = 1; h(25:28,5) = 1;
h(39:41,5) = 1; h(45:56,5) = 1; h(68:85,5) = 1;

h(1:8,6) = 1; h(25:28,6) = 1; h(42:44,6) = 1;
h(45:56,6) = 1; h(57:67,6) = 1; h(68:85,6) = 1;

writematrix(h,'h.dat','Delimiter',' ');

end
```

test_run_1.m:

```
function [ret_info,ind_stg_eq,pop_stg_eq] = test_run_1 ()

%
% Simulation of collective behavior of social distancing
%
% (Large, multi-populations)
%
% M subpopulations, M = 6
% m # individuals in subpopulations, M x 1
%
% m(1) = 250, m(2) = m(3) = m(4) = m(5) = m(6) = 120
%
% A -- connectivity matrix of social activities, n x n, n = 85
% w -- contact weights assigned to social activities, n x 1
% h -- activity sets for subpopulations, n x M
%
% ind_stg_in -- initial strategies of individuals, n x 250 x M
% pop_stg_in -- initial strategy of population, n x M
%
% ind_stg_eq -- equilibrium strategies of individuals, n x 250 x M
% pop_stg_eq -- equilibrium strategy of population, n x M
%
% ret_info -- 1 -- suceeds, 0 -- fails
%
% Zhijun Wu, 12/20/2020, Math Dept, Iowa State University
%

% Load contact matrix and contact weights

A = load('A.dat','-ascii');
w = load('w.dat','-ascii');
h = load('h.dat','-ascii');

n = size(A,1);

% Obtain # strategies and # individuals

m(1) = 250; m(2) = 120; m(3) = 120;
m(4) = 120; m(5) = 120; m(6) = 120;

M = 6;

ind_stg_in = zeros(n,250,M);
pop_stg_in = zeros(n,M);

% Start with initial random strategies
```

```
rng ('default');

for k = 1 : 10          % Repeat with different initials

   for l = 1 : M
      ind_stg_in(1:n,1:m(l),l) = rand(n,m(l));
   end

   for l = 1 : M
      for j = 1 : m(l)
         ind_stg_in(1:n,j,l) = ind_stg_in(1:n,j,l) .* h(1:n,l);
         ind_stg_in(1:n,j,l) = ind_stg_in(1:n,j,l) / sum(ind_stg_in(1:n,j,l));
      end
   end

   for l = 1 : M
      for i = 1 : n
         pop_stg_in(i,l) = sum(ind_stg_in(i,1:m(l),l)) / m(l);
      end
   end

   % Start simulation, to reach equilibrium strategies

   [ind_stg_eq,pop_stg_eq] = soc_dis_sim (ind_stg_in,pop_stg_in,A,w,h,m);

   dlmwrite(['pop_stg_eq_',num2str(k),'.dat'],pop_stg_eq,'precision','%8.6f');

end  % Repeated with different initials

ret_info = 1;

end
```

soc_dis_sim.m:

```
function [ind_stg_eq,pop_stg_eq] = soc_dis_sim (ind_stg,pop_stg,A,w,h,m)

%
% Simulation of collective behavior of social distancing
%
% (Large, multi-populations)
%
% M subpopulations, M = 6
% m # individuals in subpopulations, M x 1
%
% m(1) = 250, m(2) = m(3) = m(4) = m(5) = m(6) = 120
%
% A -- connectivity matrix of social activities, n x n, n = 85
% w -- contact weights assigned to social activities, n x 1
% h -- activity sets for subpopulations, n x M
%
% ind_stg -- strategies of individuals, n x 250 x M
% pop_stg -- strategy of population, n x M
%
% ind_stg_eq -- equilibrium strategies of individuals, n x 250 x M
% pop_stg_eq -- equilibrium strategy of population, n x M
%

% Zhijun Wu, 12/20/2020, Math Dept, Iowa State University
%

W = diag(w);
A = (A*W + W*A) / 2;

n = size(ind_stg,1);

ind_stg_eq = ind_stg;
pop_stg_eq = pop_stg;

M = size(m,1);

for l = 1 : M

   g(l) = figure;

   plot(ind_stg_eq(1:n,1:M(l),l),'ob','MarkerSize',4);
   hold;

   plot(pop_stg_eq(1:n,l),'*r','MarkerSize',4);

   title('Generation 0','FontSize',16);
```

```
   xlabel('Social Activities','FontSize',16,'FontWeight','Bold');
   ylabel('Participating Frequencies','FontSize',16,'FontWeight','Bold');

   hold;
end

pause;

% Averaged RMSD

% Initial and maximum # iterations

K = 400;

d = zeros(K,M);

% Max payoff difference

k = 0; con_max_0 = 1;

while (con_max_0 > 1.0e-12 && k < K)

  con_max_0 = 0;

  for l = 1 : M

  for j = 1 : m(l)

      x = ind_stg_eq(1:n,j,l);
      y = pop_stg_eq(1:n,l);

      act_set = h(1:n,l);

      ind_stg_eq(1:n,j,l) = soc_dis_upd(x,y,pop_stg_eq,act_set,A);

      pop_stg_eq(1:n,l) = y + (ind_stg_eq(1:n,j,l) - x) / m(l);

      con_pop = (A*sum(pop_stg_eq,2)).*act_set;

      con_ind_eq(j,l) = ind_stg_eq(1:n,j,l)'*con_pop;
      con_pop_eq(l) = pop_stg_eq(1:n,l)'*con_pop;

      con_rel_eq = con_pop_eq(l) * act_set - con_pop;

      con_ave = norm (con_rel_eq);

      if (con_ave > con_max_0)
         con_max_0 = con_ave;
```

```
      end

    end

    end

    k = k + 1;

    if (mod(k,40) == 0)

    for l = 1 : M

    figure(g(l));

    plot(ind_stg_eq(1:n,1:m(l),l),'ob','MarkerSize',4);
    hold;

    plot(pop_stg_eq(1:n,l),'*r','MarkerSize',4);

    title(['Generation ',num2str(k)],'FontSize',16);
    xlabel('Social Activities','FontSize',16,'FontWeight','Bold');
    ylabel('Participating Frequencies','FontSize',16,'FontWeight','Bold');

    hold;

    end

    pause;

    end

    for l = 1 : M

    e = ones(m(l),1);
    c = sqrt(sum((ind_stg_eq(1:n,1:m(l),l) - pop_stg_eq(1:n,l)*e').^2));
    c = c';

    d(k,l) = sum(c) / m(l);

    end

  end

  pause;

  K = k;

  for l = 1 : M
```

```
  figure(g(l));

  plot(d(1:K,l),'-b','LineWidth',2);
  hold;

  xdata = 1:1:K; xdata = xdata';
  ydata = d(1:K,l);

  x0 = [1;1;50;3];
  x1 = lsqcurvefit(@myfun,x0,xdata,ydata);
  ydata = myfun(x1,xdata);

  plot(xdata,ydata,'-r','LineWidth',4);

  title('Average Deviations of Individual Strategies','FontSize',16);
  xlabel('Generations','FontSize',16,'FontWeight','bold');
  ylabel('Average Deviations','FontSize',16,'FontWeight','bold');

  hold;

end

  pause;

  close all;

end

function ydata = myfun(x,xdata)

ydata = -x(1)*atan(x(2)*(xdata - x(3))) + x(4);

end
```

soc_dis_upd.m:

```
function ind_stg_out = soc_dis_upd (ind_stg_in,pop_stg_in,pop_stg_all_in,act_set,A)

%
% Update of individual distancing strategy
%
% M subpopulations, M = 6
% m # individuals in subpopulations, M x 1
% m(1) = 250, m(2) = m(3) = m(4) = m(5) = m(6) = 120
%
% A -- weighted connectivity matrix of social activities, n x n, n = 85
% act_set -- activity set for current individual, n x 1
%
% ind_stg_in -- current individual strategy, n x 1
% pop_stg_in -- current subpopulation strategy, n x 1
% pop_stg_all_in -- current strategies of all subpopulations, n x M

% ind_stg_out -- updated individual strategy, n x 1
%
% Zhijun Wu, 12/20/2020, Math Dept, Iowa State University
%

n = size(ind_stg_in,1);

x = ind_stg_in;
y = pop_stg_in;

con_max = (A*sum(pop_stg_all_in,2)).*act_set;
con_ave = y' * con_max;         % ave contacts for current subpop

con_rel = con_ave * act_set - con_max;

for i = 1 : n

  if (act_set(i) == 1)

  %strategy i has lower contact, increase its frequency
  if (con_rel(i) > 0)
    if (x(i) < y(i))
      x(i) = x(i) + 1.0 * (y(i) - x(i));
    else
      x(i) = x(i) + 0.5 * min(x(i)-y(i),1.0-x(i));
    end
  end

  %strategy i has higher contact, reduce its frequcy:
  if (con_rel(i) < 0)
```

```
      if (x(i) > y(i))
        x(i) = x(i) - 1.0 * (x(i) - y(i));
      else
        x(i) = x(i) - 0.5 * min(y(i)-x(i),x(i)-0.0);
      end
    end

    end

end

ind_stg_out = x / sum(x);

end
```
